# Synergy of Ag and AgBr in a Pressurized Flow Reactor for Selective Photocatalytic Oxidative Coupling of Methane

**DOI:** 10.1021/acscatal.2c06093

**Published:** 2023-03-02

**Authors:** Chao Wang, Xiyi Li, Yifei Ren, Haimiao Jiao, Feng Ryan Wang, Junwang Tang

**Affiliations:** † Department of Chemical Engineering, University College London, London WC1E 7JE, U.K.; ‡ Industrial Catalysis Center, Department of Chemical Engineering, Tsinghua University, Beijing 100084, China

**Keywords:** oxidative coupling of methane, pressurized flow reactor, synergy of Ag and AgBr, ethane, photocatalysis

## Abstract

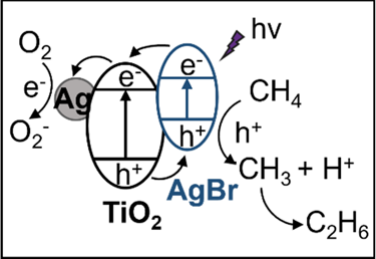

Oxidation of methane into valuable chemicals, such as C_2+_ molecules, has been long sought after but the dilemma between high yield and high selectivity of desired products remains. Herein, methane is upgraded through the photocatalytic oxidative coupling of methane (OCM) over a ternary Ag–AgBr/TiO_2_ catalyst in a pressurized flow reactor. The ethane yield of 35.4 μmol/h with a high C_2+_ selectivity of 79% has been obtained under 6 bar pressure. These are much better than most of the previous benchmark performance in photocatalytic OCM processes. These results are attributed to the synergy between Ag and AgBr, where Ag serves as an electron acceptor and promotes the charge transfer and AgBr forms a heterostructure with TiO_2_ not only to facilitate charge separation but also to avoid the overoxidation process. This work thus demonstrates an efficient strategy for photocatalytic methane conversion by both the rational design of the catalyst for the high selectivity and reactor engineering for the high conversion.

## Introduction

Large reserves of natural gas and shale gas, especially those in remote areas, have raised incentives for the on-site and large-scale conversion of methane (CH_4_) to high-value chemicals, which also avoids adverse environmental impact due to the nearly 30-time more potent greenhouse gas effect of methane than CO_2_.^1,2^ However, the low polarisability and high C–H bond energy (439 kJ/mol) of CH_4_ make its economic conversion extremely challenging.^3^ Methane conversion, including the nonoxidative coupling of methane, oxidative coupling of methane (OCM), and partial oxidation of methane, has been developed in thermocatalysis for the production of value-added products, such as C_2+_ hydrocarbons and alcohols.^4−6^ However, most of the processes require strong oxidants (H_2_O_2_ or H_2_SO_4_) and/or harsh reaction conditions (e.g., high temperature and pressure).^4,7,8^

Photocatalysis uses the energy of photons instead of heat to drive thermodynamically nonspontaneous reactions, such as water splitting, carbon dioxide reduction, etc. Since photons are the main energy source, photocatalytic reactions can be conducted under very mild conditions. Methane oxidation by oxygen gas to C_1_ oxygenates (e.g., CH_3_OH, CH_3_OOH, and HCHO) in the presence of water has been well-studied using oxide-based photocatalysts. The selectivity of products can be manipulated via the modification of different co-catalysts.^9^ For instance, a high primary products (CH_3_OOH and CH_3_OH) yield of 25.4 μmol/h and a selectivity of 95% were achieved over TiO_2_ modified by Au-CoO_*x*_ dual co-catalyst.^10^ Up to now, photocatalytic methane conversion has already been achieved over TiO_2_, ZnO, WO_3_, etc.^11−17^ However the upgrade of methane into C_2_ products is still one of the most challenging pathways as it is difficult to minimize overoxidation while maintaining a high conversion rate.^18−21^ Recently, CH_4_ was successfully converted into C_2_H_6_ at a selectivity of 90% in a photochemical looping process by an Ag-HPW/TiO_2_ photocatalyst.^22^ However, the ethane yield (2.3 μmol/h) was very moderate, and a subsequent catalyst recovery process was required to regenerate the active silver species on TiO_2_. More importantly, when O_2_ was introduced into the reaction atmosphere, only overoxidation products (CO_*x*_) were obtained.^23^

Apart from the selection of photocatalysts, the reaction system is equally important for an efficient photochemical process. Most of the reported reactors used in photocatalytic methane conversion were batch reactors.^24^ However, the products in a batch reactor easily undergo overoxidation in the presence of oxidants because all products from methane conversion are more reactive than methane itself. Thus, the use of flow reactors in photocatalytic methane conversion is crucial to manipulate the mass transfer, thus minimizing the drawback of batch reactors and improving the selectivity of the less stable valuable chemicals. Our group reported the first photocatalytic OCM in a flow reaction system, an improved ethane (C_2_H_6_) yield of 6.8 μmol/h was achieved, but it was still quite moderate.^19^ Very recently, Au-ZnO/TiO_2_ was also reported for photocatalytic OCM in a flow reactor under atmospheric pressure.^25^ A high C_2_H_6_ yield of 100 μmol/h was obtained without external heating, although the temperature of the catalyst reached 413 K due to Xe lamp irradiation. These results indicate it is still challenging to achieve a high yield of C_2_ products at low temperatures. Moreover, the reaction pressure, as a crucial factor in gas phase reactions, has not been investigated in flow systems for photocatalytic methane conversion. Considering the high pressure of natural gas in both production sites and transportation pipelines, it is economical to convert methane in pressurized reactors.

Herein, we report the selective photocatalytic OCM in a pressurized flow reactor over an Ag–AgBr/TiO_2_ catalyst. The ethane production rate of 35.4 μmol/h was achieved, together with an excellent C_2+_ selectivity of 79% operated at a low temperature of 40 °C. The utilization of a pressurized flow reactor likely enhanced the mass transfer of both reactants and products. Ag serves as an electron sink, while AgBr forms a heterostructure with TiO_2_, which improves charge separation and migration, and more importantly avoids overoxidation. Overall, the results suggest that both the photocatalyst and the reaction system play important roles in photocatalytic methane conversion.

## Results and Discussion

**Figure 1:**
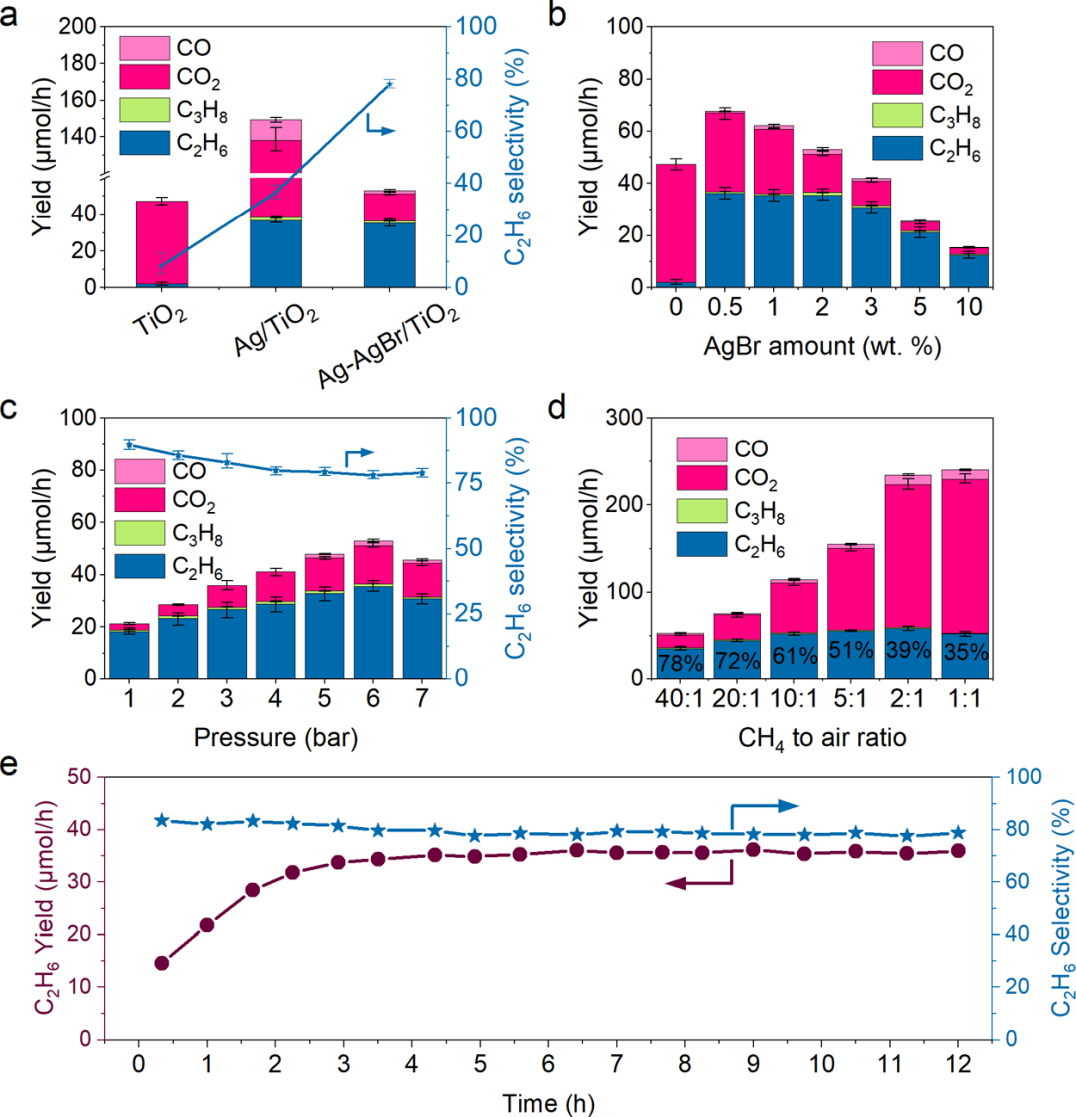
Photocatalytic oxidative coupling of methane: (a) products yield and C_2_H_6_ selectivity over TiO_2_, Ag/TiO_2_, and Ag–AgBr/TiO_2_; (b) products yield over TiO_2_ modified by different amounts of AgBr (based on wt % of metallic Ag); (c) products yield and C_2_H_6_ selectivity over Ag–AgBr/TiO_2_ under different pressures; (d) influence of CH_4_ to air ratios on the yield under 6 bar over Ag–AgBr/TiO_2_; and (e) long-term C_2_H_6_ production rate and selectivity under 6 bar pressure over Ag–AgBr/TiO_2_. Reaction conditions: flow rates of CH_4_, Air, and Ar are 40, 1, and 360 mL/min, respectively, 6 bar (except c), 40 °C, 365 nm LED, and 100 mg photocatalysts. The error bar was obtained by carrying out three tests under identical reaction conditions.

Ag and AgBr were loaded on anatase TiO_2_ by a two-step precipitation–photodeposition method as detailed in the Materials Synthesis (Supporting Information) and denoted Ag–AgBr/TiO_2_. The same amount of Ag was also loaded on TiO_2_ by photodeposition as a reference and denoted Ag/TiO_2_. The photocatalysts were tested in a pressurized flow reaction system (Scheme S1). The control experiment shows that the photocatalyst, CH_4_, and light irradiation are all indispensable to converting methane at low temperatures (Figure S1). Then, CH_4_ conversion was evaluated on TiO_2_. Bare TiO_2_ produces CO_2_ with a selectivity of 91% at a rate of 45.1 μmol/h (Figures 1a and S2). With 2 wt % Ag deposited on TiO_2_, the C_2_H_6_ production rate increases from 2.1 μmol/h to 36.7 μmol/h. Additionally, C_3_H_8_ is also produced at a rate of 1.9 μmol/h. Ag loading can facilitate the formation of C_2+_ products. However, severe overoxidation is also observed as the CO_2_ production rate surges to 99.5 μmol/h, corresponding to a selectivity of 52%. When Ag and AgBr were co-loaded on TiO_2_, the C_2_H_6_ and C_3_H_8_ production rates slightly reduce to 35.4 and 1.1 μmol/h, respectively, while CO_2_ production is substantially suppressed. A high C_2+_ selectivity of 79% has been achieved over Ag–AgBr/TiO_2_ in contrast to 8% over TiO_2_ and 44% over Ag/TiO_2_.

Following this, a series of Ag–AgBr/TiO_2_ photocatalysts with various Ag loading amounts were synthesized to optimize the photocatalytic performance under the reaction pressure of 6 bar (Figures 1b and S2). The selectivity shifts toward C_2_H_6_ (70%) even with a small AgBr amount of 0.5 wt %. Increasing the amount of AgBr to 2 wt % has little effect on the yield of C_2_H_6_ but effectively decreases the production of CO_2_. Although further increasing the AgBr amount can improve C_2+_ selectivity to as high as 90%, a decrease in the yield of all products is observed. The results indicate that AgBr plays an important role in controlling overoxidation during methane conversion. TiO_2_ decorated with 2 wt % AgBr was then chosen for further study to improve both the yield and selectivity of C_2_H_6_. The effect of reaction pressure on the photocatalytic OCM performance was investigated (Figures 1c and S3). The yield of C_2_H_6_ increases from 18.2 to 35.4 μmol/h as the reaction pressure elevates from 1 to 6 bar. An apparent quantum efficiency under 6 bar based on methane conversion was calculated to be 3% at 365 nm. The photocatalytic performance at 7 bar stops increasing, which is possibly caused by the limited photo-induced carriers generated by the photocatalyst. The enhanced performance under elevated pressures is mainly due to the enhanced mass transfer, which increases the adsorption of CH_4_ on the surface of photocatalysts. On the other hand, the selectivity of CO_2_ gradually increases from 9 to 16% as pressure increases. The partial pressures of both CH_4_ and O_2_ in the reaction atmosphere increase as the total pressure increases. Considering the activation of O_2_ is much easier than that of CH_4_,^26^ O_2_ reduction is improved more significantly than CH_4_ under higher pressures, resulting in the formation of excessive O_2_^–^ radicals, which contributes to overoxidation. Thus, the selectivity toward C_2+_ products decreases under higher pressures. The effect of CH_4_ to air ratio on the photocatalytic performance was next investigated at a total flow rate of 400 mL/min (Figures 1d and S4). When changing the ratio of CH_4_/air ratio from 40:1 to 1:1, the C_2_H_6_ production rate first increases from 35.4 to 58.1 μmol/h at the CH_4_/air of 2:1, and finally drops to 52.1 μmol/h when the CH_4_/O_2_ ratio reaches 1:1. The yield of CO_2_ is greatly accelerated with the increase of O_2_ proportion.

The optimized Ag–AgBr/TiO_2_ photocatalyst was further tested under 6 bar pressure to examine its long-term durability (Figures 1e and S5). The yield of all products increases in the first 3 h and becomes stable afterward. It suggests that there is an in situ activation process. It is well known that AgBr is light-sensitive and can decompose into Ag and Br_2_ upon exposure to irradiation.^27^ The enhancement of plasmonic Ag signal in the UV–vis diffuse reflectance spectrum (DRS) of Ag–AgBr/TiO_2_ after reaction for 3 h suggests that the amount of Ag in the photocatalyst increases, indicating AgBr underwent partial decomposition at this stage (Figure S6). The reduced Br 3d X-ray photoelectron spectroscopy (XPS) signal after the photocatalytic OCM reaction for 3 h and the similar intensity of the XPS peaks after running for 3 and 12 h suggest that AgBr is not fully decomposed even after the long-term irradiation (Figure S7). The main Ag species in AgBr/TiO_2_ before the photocatalytic reaction are positively charged silver (Figure S8i, e.g., AgBr), with only a small amount of metallic Ag. After the reaction for 3 h, the portion of metallic Ag increases while Ag^+^ decreases. This results from the fact that AgBr is partially decomposed into metallic Ag at the first 3 h of irradiation. The ratio of Ag to AgBr is calculated to be 1.34:1 based on the integrated area of the corresponding band after the catalyst is run for 3 h. Further prolonging the reaction time to 12 h results in a slight increase of Ag with an Ag to AgBr ratio of 1.57:1. The XPS results further confirm that AgBr almost remains and the chemical state of Ag in Ag–AgBr/TiO_2_ is hardly changed during the photocatalytic OCM reaction from 3 to 12 h. X-ray diffraction (XRD) also confirms the partial decomposition of AgBr after 3 h and the amount of AgBr is relatively stable in the subsequent 9 h (Figure S9), consistent with the reported.^28^ Photoluminescence (PL) spectra display improved separation of charge carriers in Ag–AgBr/TiO_2_ after 3 h of reaction (Figure S10). A similar luminescent property was observed in the photocatalyst after 3 and 12 h of methane conversion. Combined with the long-term photocatalytic performance, the above results reveal that AgBr is rather stable after the initial in situ activation process. Table S1 shows the performance of different reported photocatalysts for photocatalytic C_2_H_6_ production from methane, and it is clear that Ag–AgBr/TiO_2_ shows a high C_2_H_6_ production rates of 34.5 μmol/h in photocatalytic methane oxidation by air with a C_2+_ selectivity of 79% in a low reaction temperature of 40 °C.

**Figure 2:**
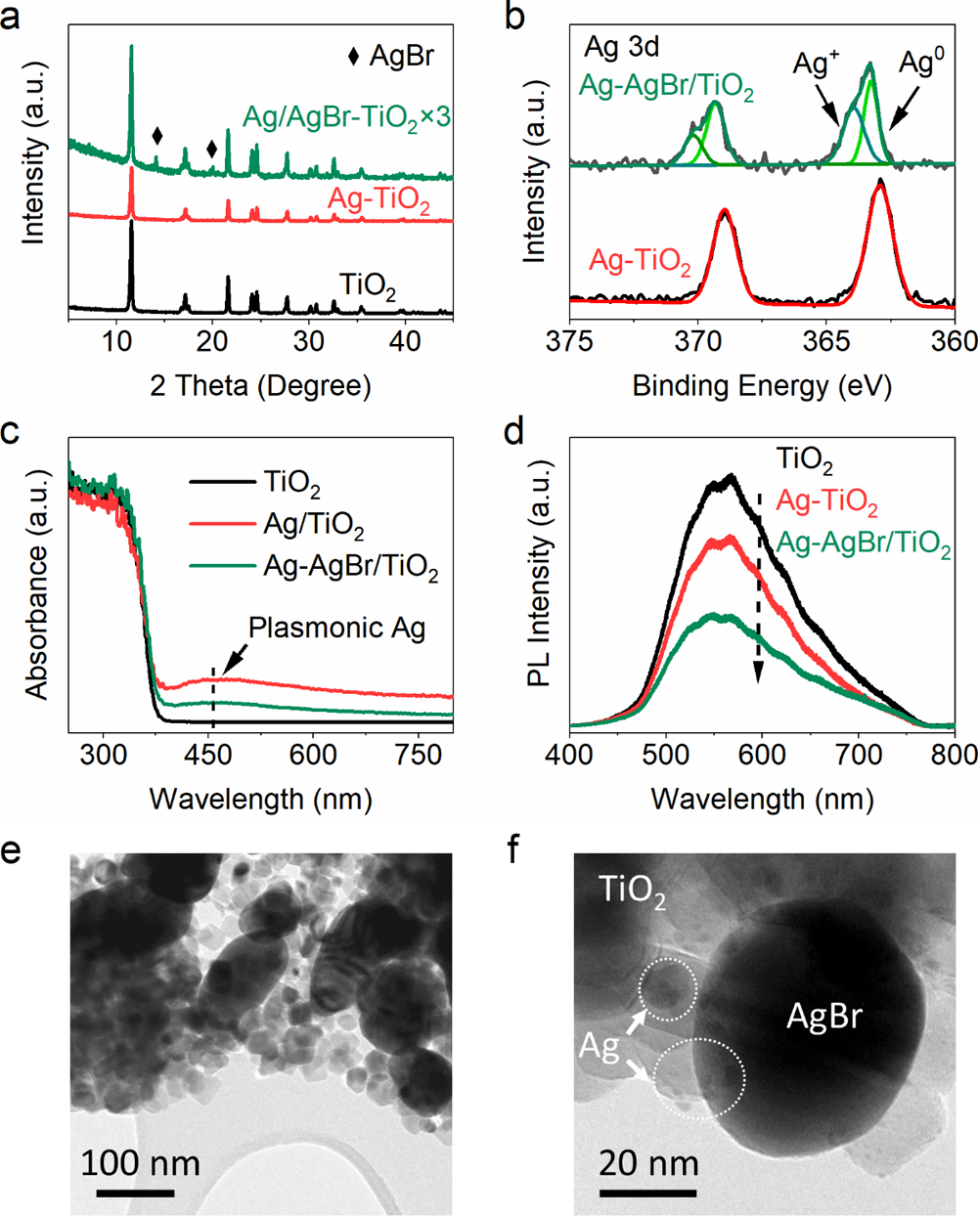
(a) XRD spectra of TiO_2_, Ag–TiO_2_, and Ag–AgBr/TiO_2_; (b) Ag 3d high-resolution XPS spectra of Ag/TiO_2_ and Ag–AgBr/TiO_2_; (c) UV–vis DRS spectra; (d) PL spectra of TiO_2_, Ag–TiO_2_, and Ag–AgBr/TiO_2_; and (e, f) TEM images of Ag–AgBr/TiO_2_.

XRD patterns of the photocatalysts display the main component of anatase (Figure 2a). Ag was not detected in either Ag/TiO_2_ or Ag–AgBr/TiO_2_, possibly due to its small particle size and/or high dispersity.^29,30^ Peaks at 14.1 and 20.0 degrees are assigned to (200) and (220) crystal planes of AgBr (PDF#06-0438), confirming the existence of AgBr on Ag–AgBr/TiO_2_. Ag 3d high-resolution XPS spectra of Ag–AgBr/TiO_2_ prove the presence of both metallic and positively charged Ag (Figure 2b). The peaks at 363.9 and 370.3 eV are attributed to the Ag 3d_5/2_ and Ag 3d_3/2_ of positively charged Ag ions, while those at 363.3 and 369.3 eV are ascribed to the Ag 3d_5/2_ and Ag 3d_3/2_ of metallic Ag.^31,32^ The molar ratio of Ag/AgBr on the surface of TiO_2_ was calculated to be 1.57:1 based on the integrated area of the related Ag 3d_3/2_ peak (or only 1.2 wt % of metallic Ag on the photocatalyst). Combined with the Br 3d spectrum (Figure S7), XPS analysis reveals the co-existence of Ag and AgBr on Ag–AgBr/TiO_2_. The band at 460 nm of UV–vis DRS spectra of Ag/TiO_2_ and Ag–AgBr/TiO_2_ is attributed to the plasmonic effect of metallic Ag (Figure 2c).^33^ Absorption of AgBr is not observed in the DRS spectrum of Ag–AgBr/TiO_2_, possibly due to the low loading amount. Then, the absorption spectrum of pure AgBr was measured (Figure S11), representing a visible absorption when the amount of AgBr is large enough. PL spectroscopy was used to investigate the charge separation and recombination process of the photocatalysts. TiO_2_ shows the highest PL emission, implying an intense recombination process. Ag loading causes a reduction in the PL intensity, and co-modification of TiO_2_ with Ag and AgBr results in the lowest PL signals. Considering the similar absorption of three catalysts in the UV region, the most efficient charge separation is achieved over Ag–AgBr/TiO_2_. To further study the effect of AgBr on charge separation and migration, the open circuit photovoltage decay spectra of three catalysts were measured (Figure S12a–c). The average lifetimes of the charges in TiO_2_, Ag/TiO_2_, and Ag–AgBr/TiO_2_ are determined to be 6.4, 12.1, and 52.9 s, respectively (Figure S12d). The eightfold increased lifetime indicates that the formation of a heterojunction between TiO_2_ and AgBr significantly prolongs the charge lifetime. The longest lifetime of charge carriers is resulted from the efficient separation of electrons and holes and could improve photon efficiency in photocatalysis.

Transmission electron microscopy (TEM) images show that TiO_2_ consists of nanoparticles of 20–30 nm (Figure S13a). High-resolution TEM (HRTEM) displays the (101) plane of anatase TiO_2_ with an interplanar spacing of 0.346 nm (Figure S13b). Metallic Ag nanoparticles can be confirmed by the line scan of Ag/TiO_2_ (Figure S14). Nanoparticles of AgBr with diameters of 100–200 nm were observed in Ag–AgBr/TiO_2_ (Figure 2e). Small Ag nanoparticles of 5 nm are also found to co-exist with AgBr on TiO_2_ in Ag–AgBr/TiO_2_ (Figure 2f). The HRTEM (Figure S15) further verifies the existence of AgBr in Ag–AgBr/TiO_2_, the interplanar distances of 0.206 and 0.290 nm are ascribed to the (220) and (200) crystal facets of AgBr, which is in accordance with the XRD analysis. In the scanning transmission electron microscopy energy-dispersive spectrometry (EDS) mapping images (Figure S16), the Ti element from TiO_2_ is detected in the selected area. Ag and Br elements are consistent with large particles of AgBr. The quality of Br mapping is slightly lower than Ag. This is mainly because that AgBr is partially decomposed under the long-term irradiation of the electron beam. Overall, Ti, O, Ag, and Br are detected in Ag–AgBr/TiO_2_ as observed from the EDS sum spectrum (Figure S16e).

**Figure 3:**
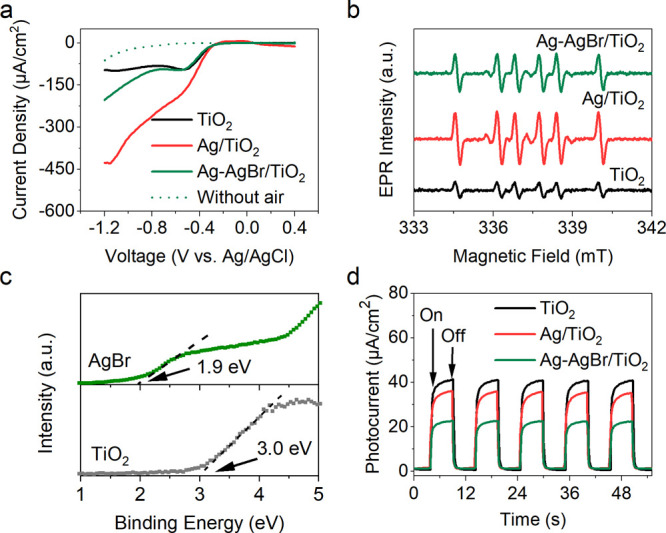
(a) Oxygen reduction LSV spectra of TiO_2_, Ag–TiO_2_, and Ag–AgBr/TiO_2_ (solid line) and LSV spectrum of Ag–AgBr/TiO_2_ tested in the absence of air (dotted line), (b) EPR O_2_^–^ spectra of TiO_2_, Ag–TiO_2_, and Ag–AgBr/TiO_2_; (c) XPS valance band spectra of TiO_2_ and AgBr; and (d) transient photocurrent plots of TiO_2_, Ag–TiO_2_, and Ag–AgBr/TiO_2_ with a bias potential of 0.25 V vs Ag/AgCl.

The reduction of oxygen gas by electrons and oxidation of methane by holes are two crucial steps during photocatalytic OCM. The oxygen reduction capability of the three photocatalysts was tested via linear sweep voltammetry (LSV) in a three-electrode cell at a potential ranging from 0.4 to −1.2 V vs Ag/AgCl (Figure S17). In the absence of air, little current is generated until the applied voltage reaches −1.0 V due to hydrogen evolution. On the contrary, a negative current is generated at the onset potential of −0.4 V in the presence of air, which is attributed to the oxygen reduction reaction. Therefore, the signal obtained in the presence of air is contributed by both oxygen reduction and hydrogen evolution. To reflect the actual oxygen reduction ability of the catalysts, the difference between the LSV spectra obtained with and without air is replotted (Figure 3a). The results show that Ag nanoparticles play a major role in oxygen reduction, as both Ag/TiO_2_ and Ag–AgBr/TiO_2_ show improved current density compared with TiO_2_ when the bias is more negative than −0.6 V. Ag/TiO_2_ exhibits the highest current density due to the highest metallic Ag amount of 2 wt %. This is consistent with the previous report that metallic Ag could promote oxygen adsorption on TiO_2_.^34^ Ag acts as an electron sink and can promote charge separation by accepting electrons from the conduction band (CB) of TiO_2_. Therefore, more photo-generated holes in the Ag-containing photocatalysts are available to activate methane molecules. As a result, the improved conversion rate of CH_4_ is achieved after the loading of Ag. O_2_ can then be reduced by electrons on the surface of Ag to produce superoxide radicals (O_2_^–^). To confirm this, the formation of O_2_^–^ radicals was monitored by electron paramagnetic resonance (EPR) using 5,5-dimethyl-1-pyrroline N-oxide as the spin-trapping reagent (Figure 3b). No EPR signal is generated in dark conditions (Figure S18), suggesting that the formation of O_2_^–^ resulted from the combination of O_2_ and photoinduced electrons. Ag/TiO_2_ and Ag–AgBr/TiO_2_ generate a higher level of O_2_^–^ radicals than TiO_2_, which remains in the same order as the LSV oxygen reduction results when the bias is more negative than −0.6 V. The highest amount of O_2_^–^ radicals are generated over Ag/TiO_2_. O_2_^–^ radicals clean the surface of the photocatalyst by combining with H^+^ to produce H_2_O. Ag serves as an electron acceptor and catalyzes O_2_ reduction, which contributes to charge separation and photon efficiency, thus resulting in improved methane conversion. However, a high level of O_2_^–^ radicals also encourage the complete mineralization of organic compounds or overoxidation to produce CO_2_.^35^ Thus, a large amount of CO_2_ (99.5 μmol/h) was detected in the photocatalytic OCM over Ag/TiO_2_. Ag–AgBr/TiO_2_ shows intermediate oxygen reduction properties among three photocatalysts, which is beneficial for reducing the selectivity of CO_2_ while maintaining a relatively high CH_4_ conversion.

XPS valence band (VB) spectra were measured, which shows that the relative VB potentials of AgBr and TiO_2_ are 1.9 and 3 eV, respectively (Figure 3c). Taking into account of the reported VB potential of anatase TiO_2_ is 2.9 V vs NHE,^36^ the VB potential of AgBr should be 1.8 V vs NHE. Thus, photoholes can potentially transfer from the VB of TiO_2_ to AgBr in Ag–AgBr/TiO_2_ upon light irradiation, resulting in a reduced oxidation potential. Thus, the overoxidation is suppressed and a high selectivity toward C_2_H_6_ is reasonable after the introduction of AgBr. To further evaluate the oxidation capability of the photocatalysts, the transient photocurrents of TiO_2_, Ag/TiO_2_, and Ag–AgBr/TiO_2_ were measured in a 0.5 M NaSO_4_ aqueous solution containing 10 vol % methanol. A bias potential of 0.25 V was applied in the test (Figures S19 and 3d). TiO_2_ shows a high photocurrent density of 40 μA/cm^2^, suggesting a fast electron transfer from the working electrode to the counter electrode and intensive methanol oxidation. When adding Ag to TiO_2_, the photocurrent is slightly reduced somehow. It is probably because the electrons are trapped by metallic Ag, which then reduces some intermediates from methanol oxidation. When methanol is removed from the electrolyte, Ag/TiO_2_ displays the highest photocurrent for water oxidation among the three catalysts (Figure S20). The lowest photocurrent is generated by Ag–AgBr/TiO_2_. After photogenerated holes transfer from the VB of TiO_2_ to AgBr, the oxidation potential is reduced, leading to a slow methanol oxidation process. This also mitigates the overoxidation of the produced C_2+_ to CO_2_. A much higher selectivity toward C_2+_ products has thus been achieved over Ag–AgBr/TiO_2_ compared with TiO_2_ and Ag/TiO_2_.

**Figure 4:**
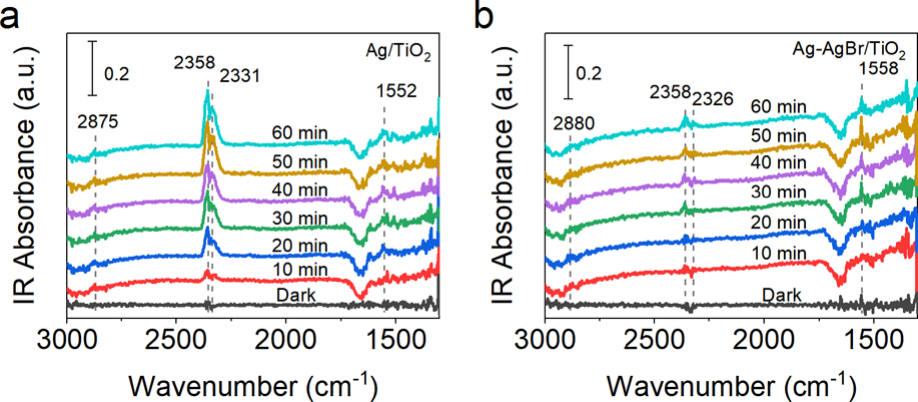
In situ DRIFTS spectra of Ag/TiO_2_ and Ag–AgBr/TiO_2_ in dark and under light irradiation in reaction atmosphere (CH_4_/air = 40:1).

To provide insights into the reaction mechanism and reaction pathway, in situ diffuse reflectance infrared Fourier transform spectroscopy (DRIFTS) was performed on Ag/TiO_2_ and Ag–AgBr/TiO_2_ (Figure 4). The infrared (IR) signal at 2875/2880 cm^–1^ under light irradiation is ascribed to the stretching vibration of C–H in CH_3_· radicals adsorbed on the oxide surface. This band is stronger in the spectrum of Ag/TiO_2_ than Ag–AgBr/TiO_2_, which shows the same trend as the methane conversion performance over the two photocatalysts. The peaks at 2358 and 2331/2326 cm^–1^ are typical signals originating from CO_2_ generated due to overoxidation in the photocatalytic OCM process. The CO_2_ peaks over Ag/TiO_2_ are much stronger than Ag–AgBr/TiO_2_ and the intensity keeps increasing with prolonged irradiation time. In contrast, Ag–AgBr/TiO_2_ generates a moderate amount of CO_2_ under identical reaction conditions. This result is in accordance with the product selectivity of Ag/TiO_2_ and Ag–AgBr/TiO_2_. An additional band at 1552/1558 cm^–1^ is ascribed to the HCOO· species, which is an important intermediate and finally results in the formation of CO_2_ in the methane oxidation process. The strong band at 1558 cm^–1^ indicates that the consumption of HCOO· is slower than its formation over Ag–AgBr/TiO_2_, suggesting a mild overoxidation process. However, the HCOO· species on Ag/TiO_2_ can be facilely converted to CO_2_, which is deduced from the low IR band intensity of HCOO· species at 1552 cm^–1^. The IR spectra over the two photocatalysts show different features when the irradiation time reaches 70 to 120 min, as displayed in Figure S21. An increased absorption across the whole spectrum is observed over Ag/TiO_2_ with the increased irradiation time. Due to the fast oxidation of methane by Ag/TiO_2_, the amount of O_2_ gas decreases rapidly in the reaction chamber. Therefore, photogenerated electrons could not be consumed due to the low level of oxygen and electrons start to accumulate on the CB of TiO_2_, consistent with the report that the photogenerated electrons display an IR absorption from 4000 to 1500 cm^–1^.^37^ Nevertheless, this phenomenon is not observed in the IR spectra of Ag–AgBr/TiO_2_. The spectra almost overlap at the irradiation time from 70 to 120 min. This indicates that a slow oxygen consumption process and a mild methane oxidation process are achieved over Ag–AgBr/TiO_2_, compared with Ag/TiO_2_. This result is in accordance with the LSV oxygen reduction and EPR O_2_^–^ trapping analysis.

To provide evidence on the universal synergy effect of Ag and AgBr on semiconductors for photocatalytic methane conversion, an Ag–AgBr/ZnO photocatalyst was synthesized by the same method, and its performance is compared with the pristine ZnO. ZnO, with a similar band structure as TiO_2_, should display similar behavior as TiO_2_ in photocatalytic methane conversion. The results are shown in Figure S22. When pure ZnO is applied as the photocatalyst, CH_3_OH and CO_2_ are generated at production rates of 6.1 and 7.5 μmol/h, respectively. It suggests that the methyl radicals formed from the reaction between methane and photoholes are mostly overoxidised into CO_2_. The production of CH_3_OH could probably be due to the unique surface features of ZnO, which might facilitate CH_3_OH desorption. Overall, coupling of methyl radicals is not encouraged on ZnO surfaces as C_2_H_6_ is not detected in the products. After modification with Ag–AgBr, C_2_H_6_ with a high production rate of 22.8 μmol/h is detected. A trace amount of C_3_H_8_ (0.4 μmol/h) is also produced, which is the product of further C_2_H_6_ activation. CH_3_OH is not detectable in the products. Most importantly, the production rate of CO_2_ is reduced from 7.5 to 4.2 μmol/h after the modification of ZnO with AgBr. Combined with the methane oxidation performance of the Ag–AgBr/TiO_2_ photocatalyst, direct evidence of the function of AgBr in facilitating C_2_H_6_ production and reducing overoxidation is obtained.

**Scheme 1:**
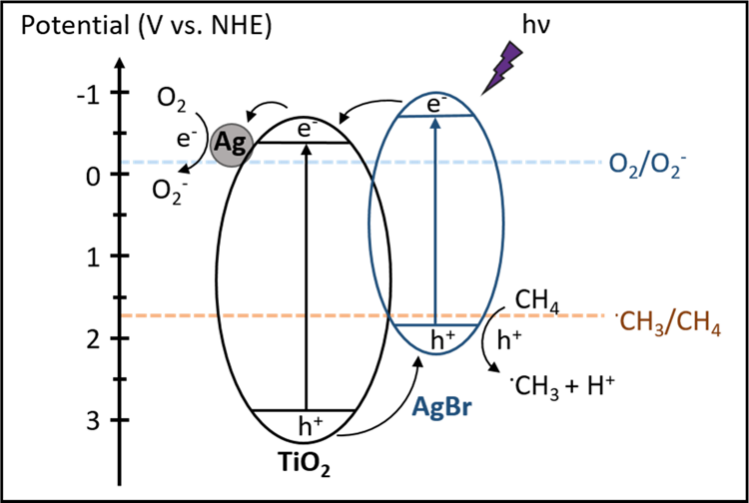
Photocatalytic Reaction Pathway of Ag–AgBr/TiO_2_

The band gaps of TiO_2_ and AgBr were determined to be 3.2 and 2.5 eV, respectively, from the Kubelka–Munk conversion plots (Figure S23), consistent with the reported.^36^ Combined with the XPS VB analysis (Figure 3c), the CB potentials of AgBr and TiO_2_ are determined to be −0.7 and −0.3 V vs NHE, respectively. In Ag–AgBr/TiO_2_, AgBr forms a type II heterojunction with TiO_2_ (Scheme 1). Upon light irradiation, photoelectrons tend to transport from the CB of AgBr to TiO_2_, and further to Ag, while photoholes transfer from the VB of TiO_2_ to AgBr. Considering the potentials required for O_2_^–^ formation from O_2_ reduction and ·CH_3_ production from CH_4_ oxidation are −0.16 and 1.75 V vs NHE,^1,22,38^ such a structure is not only beneficial for charge separation but also capable of driving the photocatalytic OCM reaction, therefore resulting in improved photon utilization efficiency and a high methane conversion. Although the enhanced charge separation is also achieved in Ag/TiO_2_, the highly oxidative holes at the VB of TiO_2_ and the large number of O_2_^–^ radicals formed on the metallic Ag cause severe overoxidation and significantly deteriorate the selectivity toward C_2+_ products (Scheme S2 in the Supporting Information). After the introduction of AgBr, photogenerated holes at the VB of TiO_2_ transfer to that of AgBr and then oxidize methane into methyl radicals and protons. A mild methane oxidation process is thus achieved by the less oxidative holes at the VB of AgBr. Next, the formed methyl radicals are prone to couple into C_2_H_6_ and are less likely to undergo overoxidation due to the relatively weak oxidation potential of photoholes in the VB of AgBr. In parallel, O_2_^–^ radicals react with protons to generate water. Some C_2_H_6_ molecules are activated again by photoholes to form ·C_2_H_5_ radicals, which couple with ·CH_3_ radicals and form C_3_H_8_.

## Conclusions

In summary, an efficient and selective photocatalytic OCM process has been realized with a ternary Ag–AgBr/TiO_2_ photocatalyst in a pressurized flow reactor. The production rate of C_2_H_6_ achieved is as high as 35.4 μmol/h, with a C_2+_ selectivity of 74–90% depending on the pressures used, together with an apparent quantum efficiency of 3% at 365 nm. These results suggest that both the reaction system and the photocatalyst play important roles in the performance of photocatalytic methane conversion as detailed by a series of characterizations. The utilization of a pressurized flow reactor enhances the mass transfer of reactants and products, contributing to the high methane conversion and selectivity toward C_2+_ products. A series of electrochemical tests and EPR results proved that Ag nanoparticles serve as an electron acceptor to improve charge separation, while the reactive holes from TiO_2_ transfer to AgBr and become less oxidative to avoid overoxidation. Therefore, both the high yield and high selectivity of C_2+_ products have been obtained. The findings demonstrate a potential to realize the efficient and selective conversion of methane to C_2+_ by the synergy of Ag and AgBr driven by photocatalysis.

## Supporting Information Available

Experimental procedures and characterization data (PDF)
