# Supplementary material for: Synergy of Ag and AgBr in a Pressurized Flow Reactor for Selective Photocatalytic Oxidative Coupling of Methane
Source: ACS Catal. 2023 Mar 2;13(6):3768–74. doi: 10.1021/acscatal.2c06093 (PMC10028603; doi:10.1021/acscatal.2c06093)
Supplement: Supplementary file 1 — cs2c06093_si_001.pdf [file cs2c06093_si_001.pdf]

Supporting Information for

Synergy of Ag and AgBr in a pressurised flow reactor for selective photocatalytic oxidative coupling of methane

Chao Wang,† Xiyi Li,† Yifei Ren,† Haimiao Jiao,† Feng Ryan Wang,† Junwang Tang†‡*

†Department of Chemical Engineering, University College London, London WC1E 7JE, UK

‡Industrial Catalysis Center, Department of Chemical Engineering, Tsinghua University, Beijing 100084, China

*Corresponding Author- Junwang Tang, Email: junwang.tang@ucl.ac.uk

Experimental

Materials synthesis

Synthesis of Ag-AgBr/TiO_2

Ag and AgBr (overall Ag 2 wt.%) were loaded on TiO_2 by a two-step precipitation-photodeposition method. 100 mg TiO_2 (Anatase, PC50, Millennium) was dispersed in 40 mL deionised water and sonicated for 5 min. Then, 1 mL 2 mg/mL (based on metallic Ag) $AgNO_3$ solution was added to the above suspension and stirred for 30 min. Afterwards, a stoichiometric amount of (2 mg/mL) NaBr solution was added drop by drop and stirred for another 1 h, allowing the formation of AgBr on TiO_2. Subsequently, the suspension was irradiated by a 300 W Xe lamp for 20 min under stirring. The product was washed by centrifugation and dried at 60 °C. The final product was denoted Ag-AgBr/TiO_2. The ratio of Ag : AgBr over the optimised sample was determined to be 1.57:1 by XPS analysis. To optimise the photocatalytic performance, a series of photocatalysts with different AgBr loading amounts from 0.1 wt.% to 10 wt.% were also prepared following the same procedure, but with different amounts of $AgNO_3$ and NaBr solution added.

Synthesis of Ag/TiO_2

Ag (2 wt.%) was loaded on TiO_2 by a photodeposition method as a reference. 100 mg TiO_2 was dispersed in 40 mL deionised water and sonicated for 5 min. Then, 1 mL of 2 mg/mL (based on metallic Ag) $AgNO_3$ solution was added to the above suspension and stirred for 30 min. The suspension was irradiated by a 300 W Xe lamp for 20 min under stirring. The product was washed by centrifugation and dried at 60 °C. The final product was denoted Ag/TiO_2.

Characterisations

X-ray diffraction (XRD) was carried out with a Stoe STADI-P diffractometer using Mo Kα source (λ=0.7093 Å) from 5 to 45°. X-ray photoelectron spectroscopy (XPS) was measured by a Thermo Scientific XPS instrument equipped with an Al Kα source (hv=1486.6 eV). The specific surface areas of TiO_2, Ag/TiO_2 and Ag-AgBr/TiO_2 are measured to be 48, 47, and 44 m^2/g by N_2 adsorption-desorption at -196 °C. Ultraviolet-visible diffuse reflectance spectroscopy (UV-Vis DRS) was measured by a Shimadzu UV-2550 spectrophotometer fitted with an integrating sphere. The reflectance values were directly converted to absorption by the Kubelka-Munk equation via the UV-Probe 2.33 software. Photoluminescence (PL) was

collected from 330 nm to 800 nm by a Renishaw InVia spectroscopy with a 325 nm laser as the excitation source. Transmission electron microscopy (TEM) was carried out on a JEOL 2010 instrument.

Superoxide radical trapping experiment

Superoxide (O_2^-) radicals were trapped by 5,5-dimethyl-1-pyrroline N-oxide (DMPO) as the spin-trapping reagent and monitored through electron paramagnetic resonance (EPR) in a Bruker E580 X-band spectrometer. Typically, 5 mg photocatalyst was dispersed in 5 mL 10 vol.% methanol solution containing 20 mM DMPO. Then, the suspension was irradiated by a 365 nm LED for 5 min under stirring. 2 mL of the suspension was filtered to remove the solid catalyst. The obtained transparent liquid was loaded into a capillary tube. The capillary tube was quickly sealed and put into a quartz tube for measurement. The EPR measurement was conducted at room temperature, with a modulation frequency of 100 kHz, a microwave frequency of 9.46 GHz, a sweep time of 60 s, a microwave power of 10 mW, and swept from 300 to 363 mT. The experiment conducted in the dark condition is the same as above but without the 5-min LED irradiation.

Electrochemical test

The working electrode was prepared by a spin-coating method. 10 mg photocatalyst was dispersed in 1 mL ethanol and sonicated for 30 min. Then 80 µL of the suspension was dropped on a piece of Fluorine-doped Tin Oxide (FTO) glass. The FTO was fixed on the spin coater for 30 s at 500 rpm. Finally, the electrode was dried on a 120 °C hot plate. All the electrochemical tests were performed in a three-electrode cell with 0.5 M $NaSO_4$ solution. The FTO coated with the photocatalyst, a Pt plate, and a Ag/AgCl in 3 M KCl electrode were used as the working electrode, counter electrode, and reference electrode, respectively. For the oxygen reduction test, the electrolyte was purged by Ar (for tests without air) or air (for tests with air) for 20 min before each scan. The linear sweep voltammetry (LSV) was performed at a voltage window from 0.4 to -1.2 V. For the oxidation capability test, 10 vol.% of methanol was added into the electrolyte to improve signal quality. In the LSV test from -0.6 to 1 V, a scan rate of 0.5 V/s was applied. During the test, the lamp was chopped on and off automatically at an interval of 5 s. The light source used was a 150 W Xe lamp. A bias potential of 0.25 V was applied in the I-t test. Similarly, an on-and-off interval of 5 s was applied over a period of 60 s. The I-t curve was also measured in the electrolyte without methanol. For the open circuit photovoltage decay (OCVD) test, the photoelectrochemical cell was first kept in dark for 10 min to ensure an equilibrium potential was obtained. Then, the open circuit voltage was recorded. The working electrode was illuminated by a 150 W Xe lamp at Time = 50 s, after which a photovoltage is generated. The generation of photovoltage results from the accumulation of charge carriers in the photocatalysts. A negative photovoltage is obtained, as the major carriers are electrons in TiO_2. After irradiation for another 50 s, the light was turned off and fast decay of the photovoltage was observed. The decay of photovoltage is a result of the recombination of charge carriers. The decay part is fitted with a biexponential function:

$$V = A_0 + A_1 e^{-\frac{t}{\tau_1}} + A_2 e^{-\frac{t}{\tau_2}}$$

Where τ_1 and τ_2 are time constants. The average lifetime of the charge carriers (τ_m) could be calculated by:

$$\tau_m = 2 (\tau_1 \tau_2)/(\tau_1 + \tau_2).$$

In situ diffuse reflectance infrared Fourier transform spectroscopy (DRIFTS) measurement

In situ DRIFTS was measured with a Shimadzu IRTracer-100 Fourier transform infrared spectrometre equipped with a Harrick cell and a Pray Mantis accessory. Two ZnSe windows and one quartz window were used on the cell for IR transmittance and UV irradiation, respectively. In each test, 100 mg catalyst was loaded in the sample holder of the cell. A metal spatchaler was used to create a flat surface of the sample for measurement. Then, the cell was purged with CH_4, air, and Ar at flow rates of 40, 1, 359 mL/min for 30 min in dark. Afterwards, the cell was sealed and fixed in the Praying Mantis accessory. The Praying Mantis accessory with the Harrick cell was finally fixed in the FTIR spectrometre for measurement. The signal of the photocatalyst in the reaction atmosphere in dark was used as background. A 12 W 365 nm LED was used to irradiate the catalysts during measurement. The IR spectra were obtained every 10 min for 120 min for each catalyst.

Photocatalytic oxidative coupling of methane

The photocatalytic OCM test was carried out in a pressurised flow system (Scheme S1). The flow rates of gases were controlled by three mass flow controllers (Bronkhorst). Two pressure gauges were installed before and after the reactor to monitor the pressure change during the photocatalytic reaction. No detectable change in the pressure was observed. The regulator valve at the end of the reaction system was used to adjust and maintain the pressure in the reactor at a range from 1 to 7 bar. The photocatalyst was filtered onto a glass fibre membrane before the performance test. To prepare the membrane, 100 mg photocatalyst was dispersed in 50 mL deionised water and sonicated for 5 min. Then the suspension was filtered by the membrane and dried at 60 °C for 8 h. The membrane was fitted in the reactor by a stainless steel ring. The reactor was sealed with a rubber ring and a stainless steel clamp, which was equipped with a quartz window. The light source used was a 365 nm LED light (35 W, Beijing Perfect Light). The temperature of the catalyst surface was monitored to be 39-41 °C. The product released from the regulator valve was directly connected to a GC (Varian 450) equipped with a TCD detector, a methanizer, and an FID detector. The selectivities are calculated based on observable products as the following:

$$\text{Selectivity of } C_2H_6 = \frac{2\times n_{C_2H_6}}{2\times n_{C_2H_6}+3\times n_{C_3H_8}+n_{CO_2}}\times 100\%$$

$$\text{Selectivity of } C_3H_8 = \frac{3\times n_{C_3H_8}}{2\times n_{C_2H_6}+3\times n_{C_3H_8}+n_{CO_2}}\times 100\%$$

$$\text{Selectivity of } CO_2 = \frac{n_{CO_2}}{2\times n_{C_2H_6}+3\times n_{C_3H_8}+n_{CO_2}}\times 100\%$$

$$\text{Selectivity of } C_{2+} = \frac{2\times n_{C_2H_6}+3\times n_{C_3H_8}}{2\times n_{C_2H_6}+3\times n_{C_3H_8}+n_{CO_2}}\times 100\%$$

The apparent quantum efficiency (AQE) was calculated based on the conversion of methane:

$$\text{AQE} = \frac{Number\ of\ electrons\ transferred}{Number\ of\ incident\ photons}\times 100\%$$

$$= \frac{\left(2\times n_{C_2H_6}+4\times n_{C_3H_8}+8\times n_{CO_2}\right)\times N_A}{\frac{I\times A}{E_g\times J}}\times 100\%$$

Where N_A is Avogadro's constant 6.02 $\times 10^{23}$, I is the light intensity 100 mW/cm^2, A is the irradiation area 7 cm^2, E_g is the energy of a photon with 365 nm wavelength and J is the

amount of charge in one electron and used to transform the unit of photon energy from eV to J.

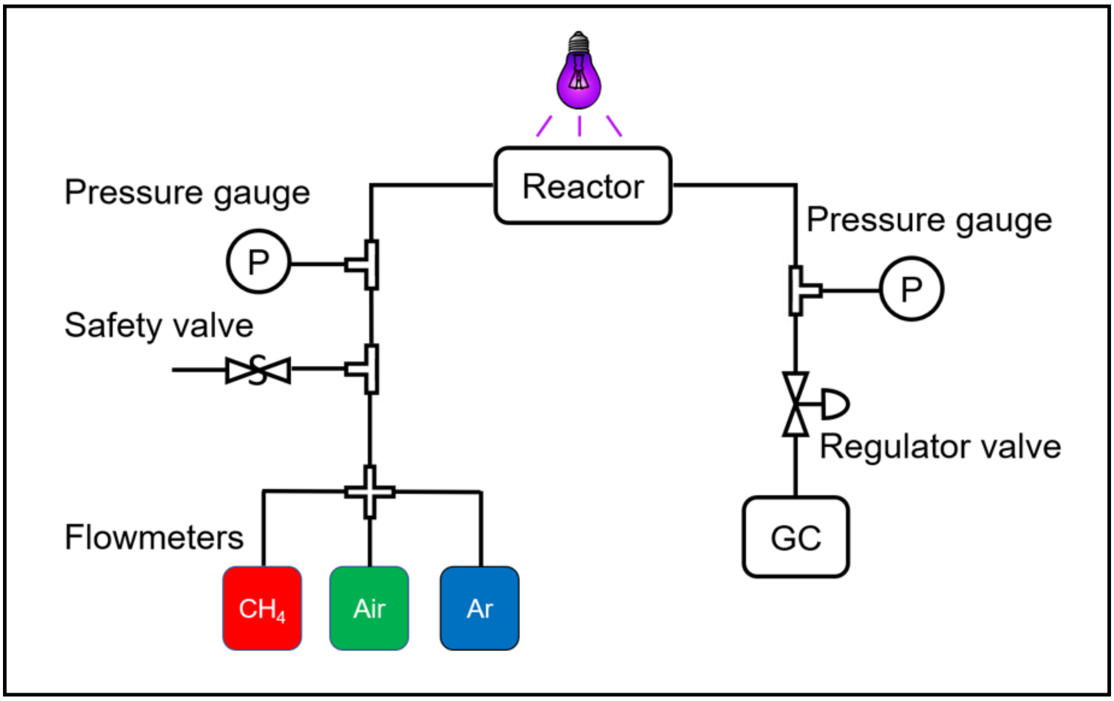

Scheme S1. A schematic illustration showing the pressurised flow reaction system for photocatalytic OCM.

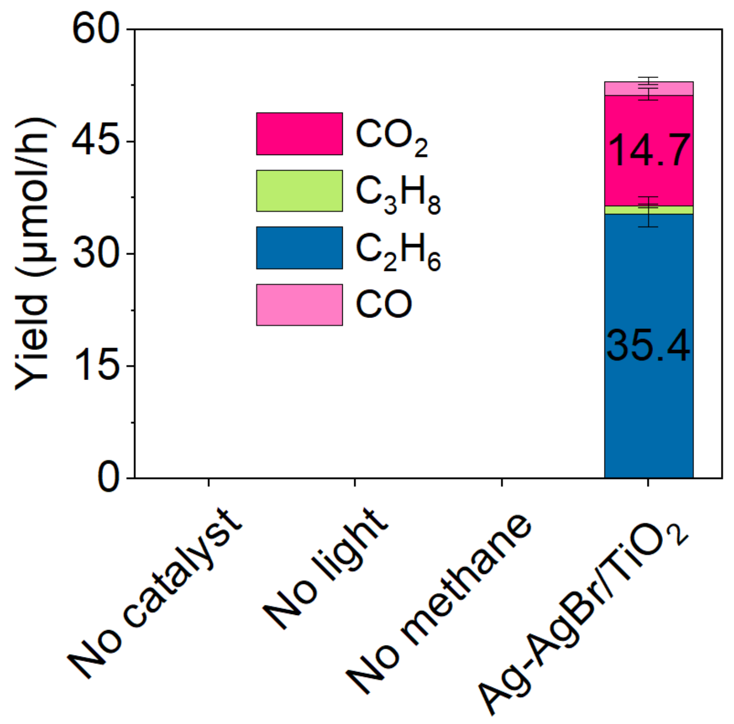

Figure S1. Product yield under conditions with no catalyst, no light, and no methane, and standard photocatalytic reaction conditions (Reaction conditions: Total flow rate 400 mL/min; 40 °C, 6 bar).

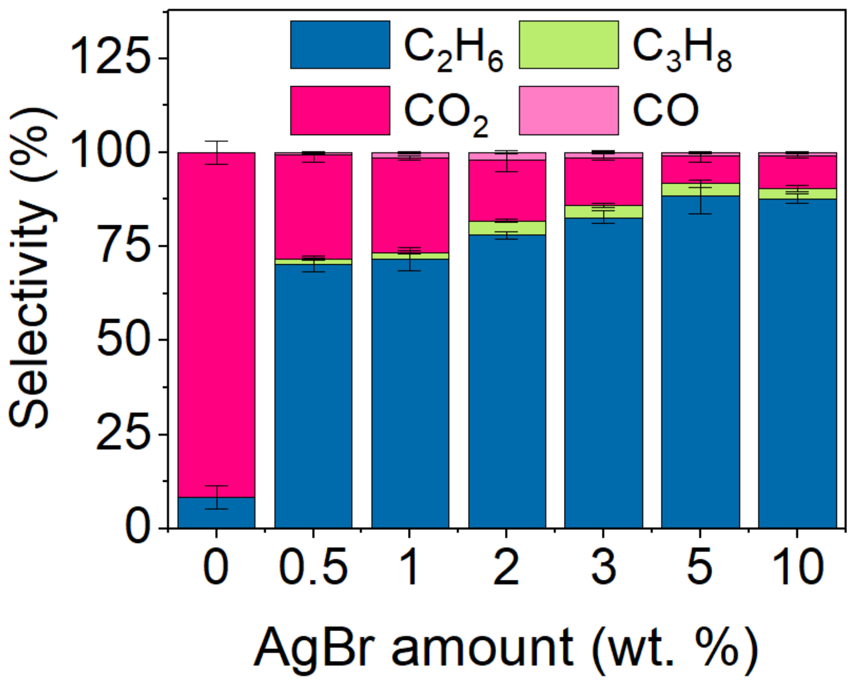

Figure S2. Product selectivity of Ag-AgBr/TiO_2 with different amounts of AgBr (Reaction conditions: flow rate of CH_4, Air, Ar=40, 2, 360 mL/min; 40 °C, 6 bar, 365 nm LED).

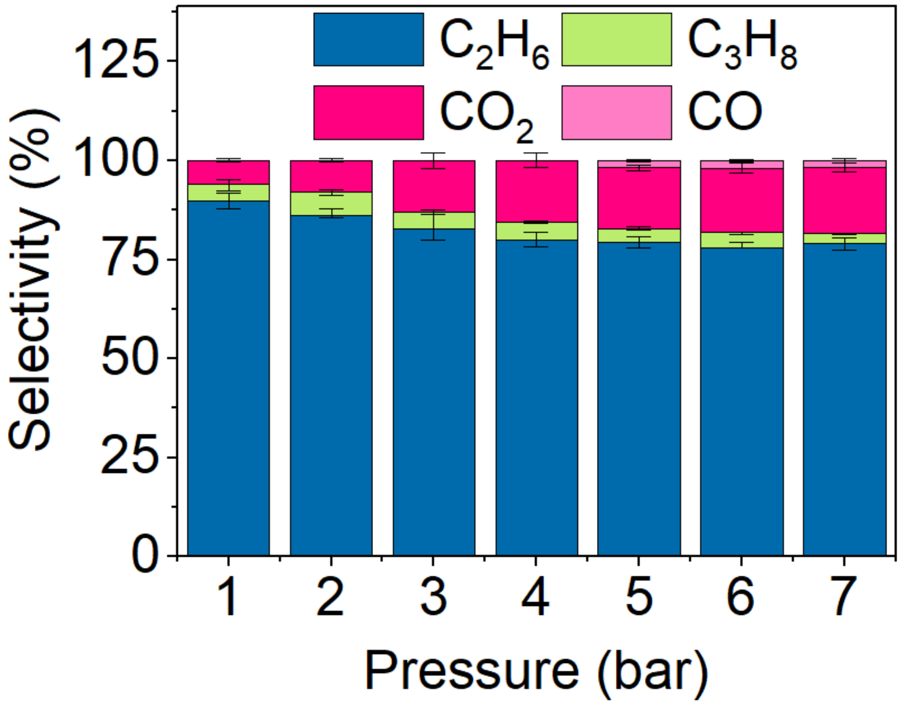

Figure S3. Product selectivity of Ag-AgBr/TiO_2 under different pressures (Reaction conditions: flow rate of CH_4, Air, Ar=40, 2, 360 mL/min; 40 °C, 365 nm LED)

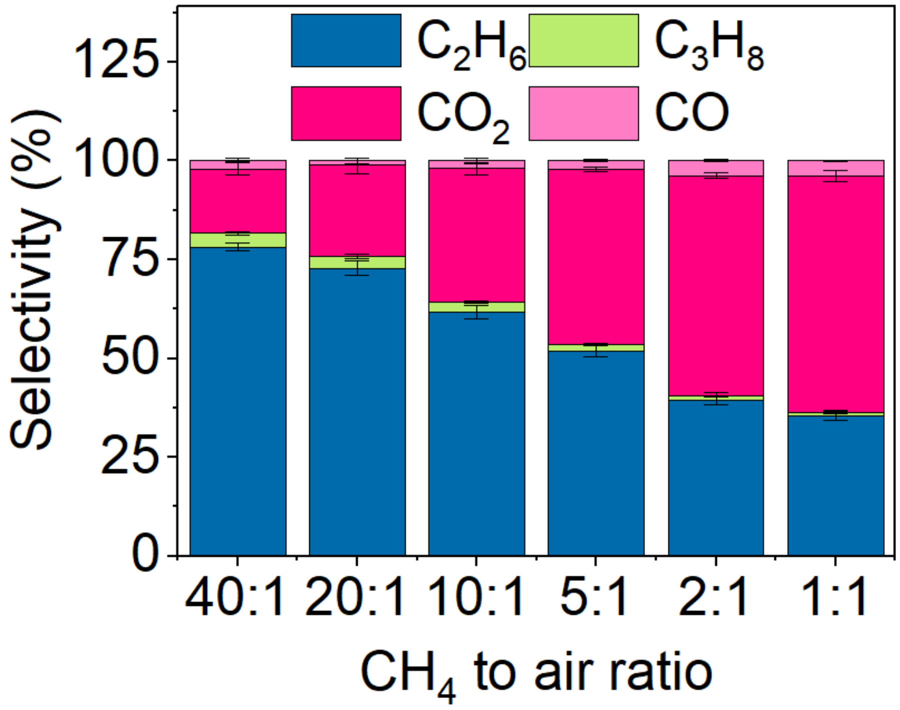

Figure S4. Product selectivity of Ag-AgBr/TiO_2 at different CH_4 to air ratios (Reaction conditions: total flow rate: 400 mL/min; 40 °C, 365 nm LED)

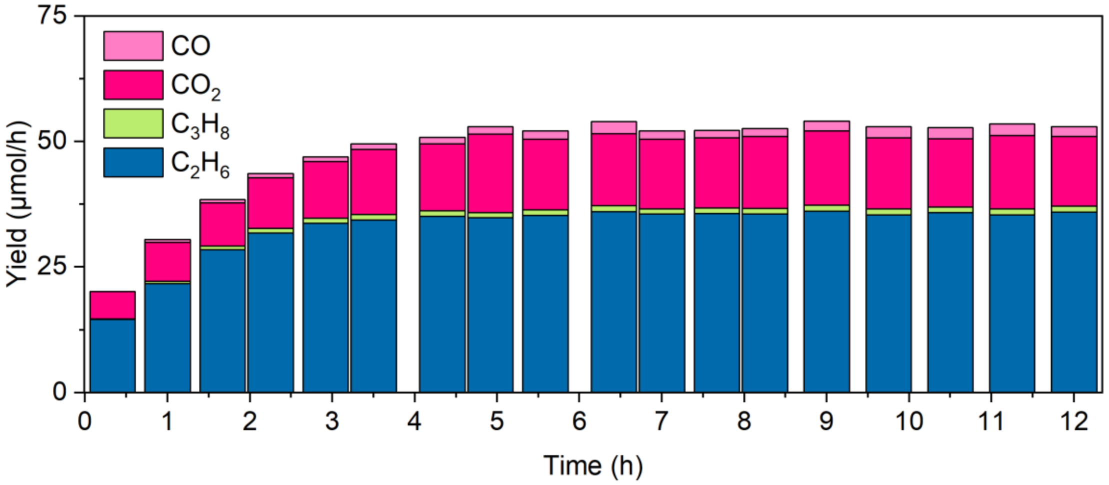

Figure S5. The long-term photocatalytic performance of Ag-AgBr/TiO_2 (Reaction conditions: flow rates of CH_4, Air, Ar=40, 2, 360 mL/min; 40 °C, 6 bar, 365 nm LED)

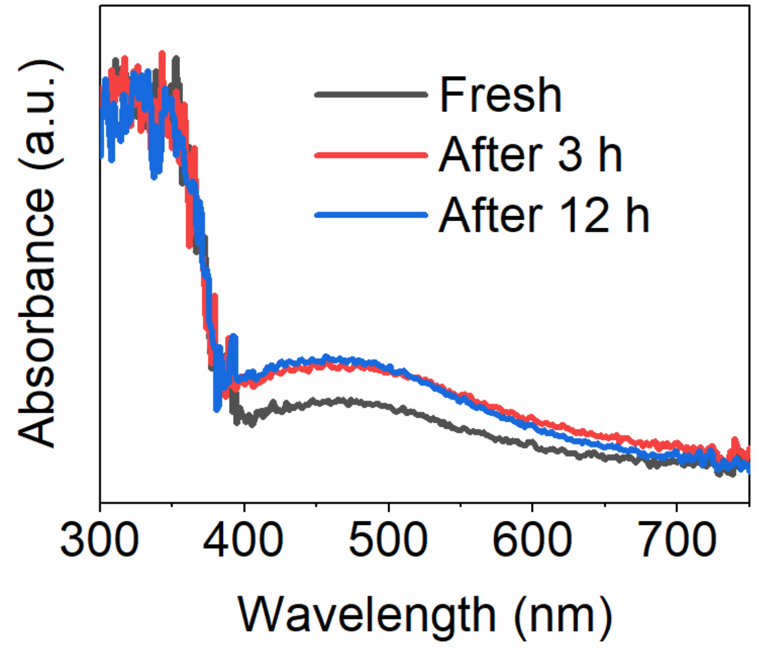

Figure S6. UV-Vis DRS spectra of fresh Ag-AgBr/TiO_2 and Ag-AgBr/TiO_2 after photocatalytic reactions for 3 h and 12 h.

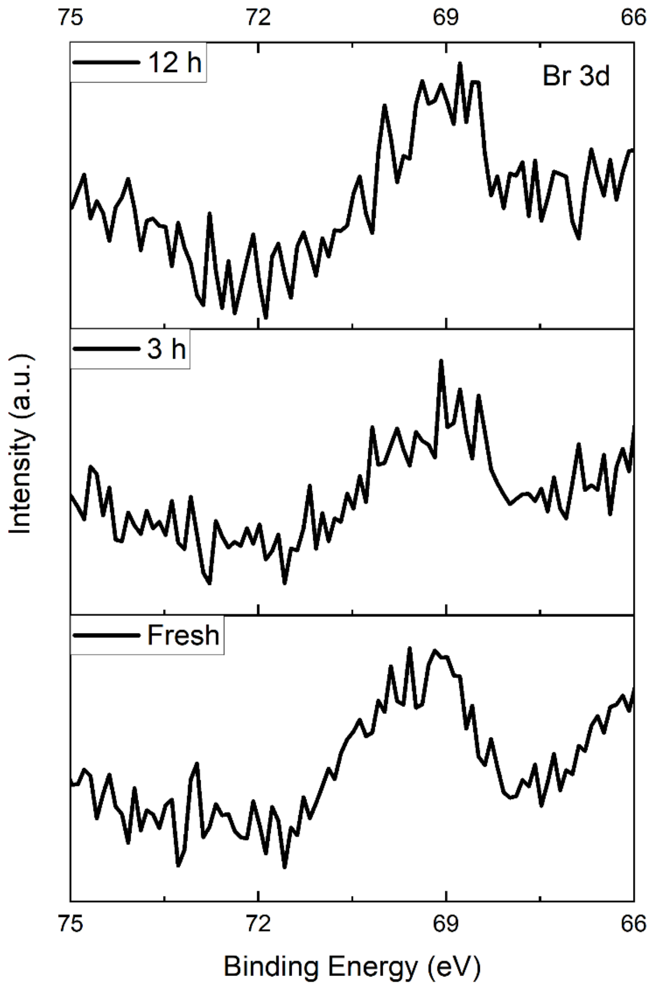

Figure S7. Br 3d spectra of Ag-AgBr/TiO_2 photocatalyst before the reaction, run for 3 h and 12 h.

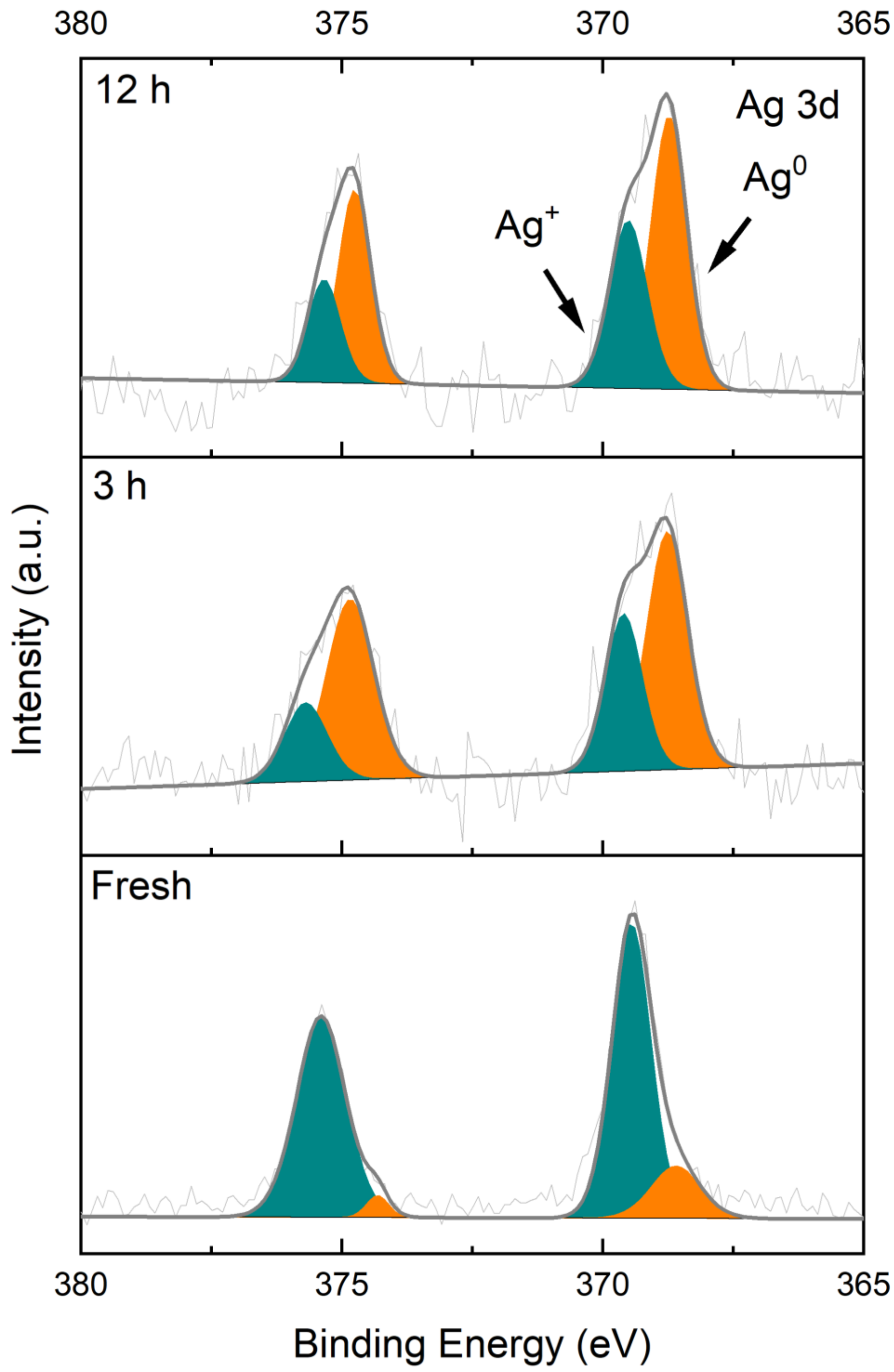

Figure S8. Ag 3d spectra of Ag-AgBr/TiO_2 photocatalyst before the reaction, run for 3 h and 12 h.

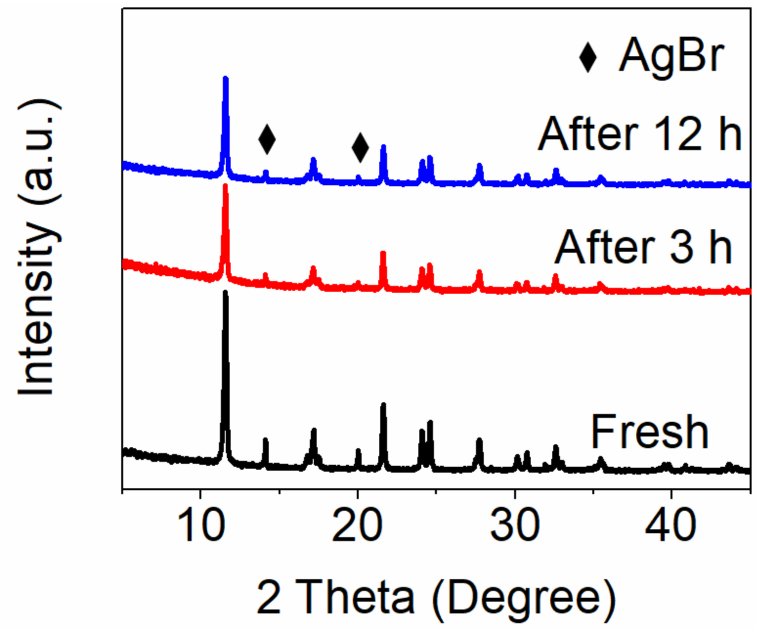

Figure S9. XRD spectra of fresh Ag-AgBr/TiO_2 and Ag-AgBr/TiO_2 after photocatalytic reactions for 3 h and 12 h.

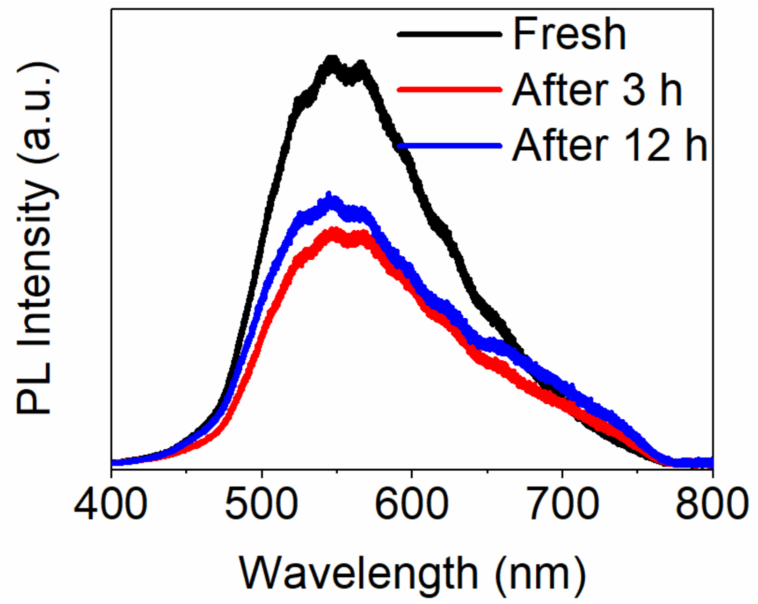

Figure S10. PL spectra of fresh Ag-AgBr/TiO_2 and Ag-AgBr/TiO_2 after photocatalytic reactions for 3 h and 12 h.

Table S1. A comparison of the photocatalytic C_2H_6 production performance of Ag-AgBr/TiO_2 with those reported in the literature.

Catalyst	Reaction Conditions	C_2H_6 Yield umol/(h·g)	C_2H_6 Yield μmol/(h·m^2)	AQE
Ag-AgBr/TiO_2 (This work)	Flow reactor, 365 nm LED, 40 °C, 6 bar, 100 mg catalyst	354	8	3% at 365 nm
Au/WO_3[1]	Batch reactor, 300 W Xe lamp, peroxymonosulfate oxidant, 20 mg catalyst	76	-	-
GaN:ZnO[2]	Batch reactor, 300 W Xe lamp, 2 mg catalyst	165	8.2	0.7% at 325 nm
Pt/TiO_2[3]	Batch reactor, 4 W 254 nm UV lamp, 75 mg catalyst	56	-	4.7% at 254 nm
Pd/TiO_2[4]	Batch reactor, 4 W 254 nm UV lamp, 75 mg catalyst	56	-	2.8% at 254 nm
Pt/Ga-TiO_2-SiO_2[5]	Batch reactor, 300 W Xe lamp, 200 mg catalyst	1.6	0.009	0.0001% at 350 nm
Ag-HPW/TiO_2[6]	Batch reactor, 400 W Xe lamp, 100 mg catalyst	21	0.74	3.5% at 365 nm
FSM-16[7]	Batch reactor, 300 W Xe lamp, 200 mg catalyst	0.1	0.0001	-
H-MOR[8]	Batch reactor, 250 W Xe lamp, 1 g catalyst	0.07	-	-
SiO_2–Al_2O_3[9]	Batch reactor, 250 W Xe lamp, 1 g catalyst	0.1	0.0001	-
SiO_2-Al_2O_3[10]	Batch reactor, 250 W Xe lamp, 1 g catalyst	0.02	0.0003	-
SiO_2-Al_2O_3-TiO_2[11]	Batch reactor, 250 W Xe lamp, 1 g catalyst	0.69	0.0003	-
ZrO_2/SiO_2[12]	Batch reactor, 250 W Xe lamp, 500 mg catalyst	0.08	-	-
TiO_2/SiO_2[13]	Batch reactor, 300 W Xe lamp, 200 mg catalyst	1	-	-
Ga_2O_3-K[14]	Batch reactor, 300 W Xe lamp, 200 mg catalyst	0.25	0.12	-

Ga-ETS-10[15]	Batch reactor, 150 W Hg lamp, 200 mg catalyst	11	0.033	-
(Zn^{+}, Zn^{2+})-ZSM-5[16]	Batch reactor, 150 W Hg lamp, 1 g catalyst	2.9	0.008	0.55% at 300-400 nm
Ce^{3+}-Al_2O_3[17]	Batch reactor, 300 W Xe lamp, 200 mg catalyst	0.25	0.0015	-
Ce-Al_2O_3[18]	Batch reactor, 300 W Xe lamp, 200 mg catalyst	0.45	0.0027	-
MgO-SiO_2[19]	Batch reactor, 300 W Xe lamp, 200 mg catalyst	0.05	0.0001	-
Au/ZnO[20]	Batch reactor, 300 W Xe lamp, 1 mg catalyst	10	-	-
AuPd/ZnO[21]	Batch reactor, 300 W Xe lamp, 2 mg catalyst	25	-	-
Pt/Ga_2O_3[22]	Flow reactor, 254 nm UV light, 3 bar, 50 mg catalyst	696	69.6	13% at 254 nm
Pd-Bi/Ga_2O_3[23]	Flow reactor, 254 nm UV light, 800 mg catalyst	1.2	0.07	0.2% at 220-300 nm
Pd/ Ga_2O_3[24]	Flow reactor, 40 W Hg lamp, 50 mg catalyst	940	104	5.1% at 254 nm
Au/TiO_2[25]	Flow reactor, 300 W Xe lamp, 5 mg catalyst	80	-	-
Pt-CuO_x/TiO_2[26]	Flow reactor, 40 W 365 nm LED, 100 mg catalyst	68	-	0.5% at 365 nm
Au-ZnO/TiO_2[27]	Flow reactor, 300 W Xe lamp, 20 mg catalyst	5000	64.1	7.2% at 360 nm

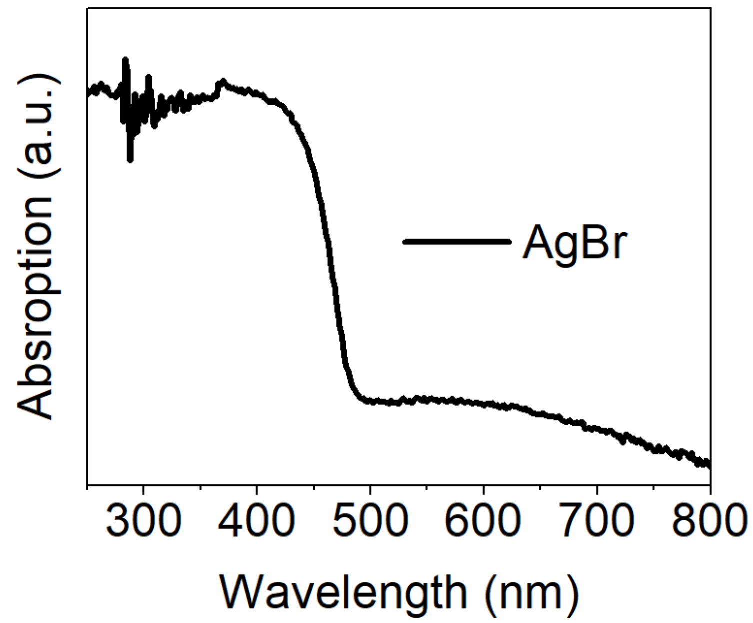

Figure S11. UV-Vis DRS spectrum of AgBr.

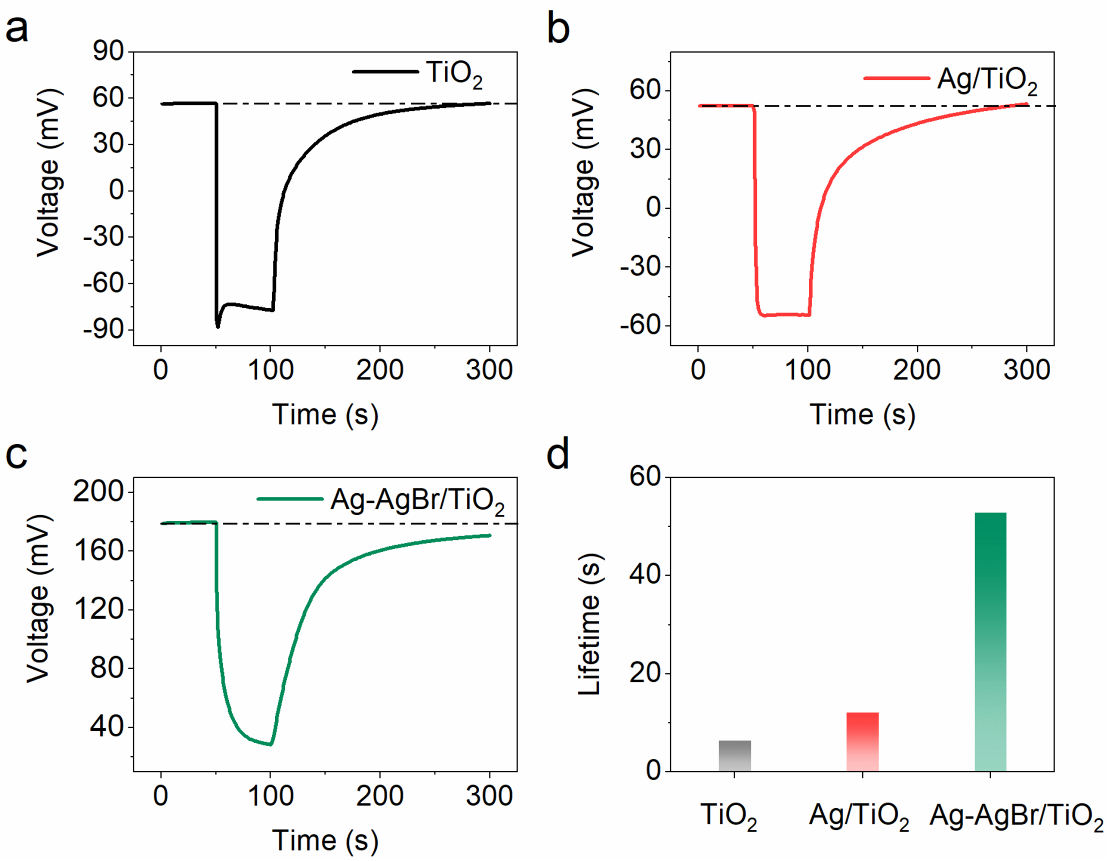

Figure S12. Open circuit photovoltage decay spectra of (a) TiO_2, (b) Ag/TiO_2 and (c) Ag-AgBr/TiO_2, (d) the calculated average lifetime of charge carriers in TiO_2, Ag/TiO_2, and Ag-AgBr/TiO_2.

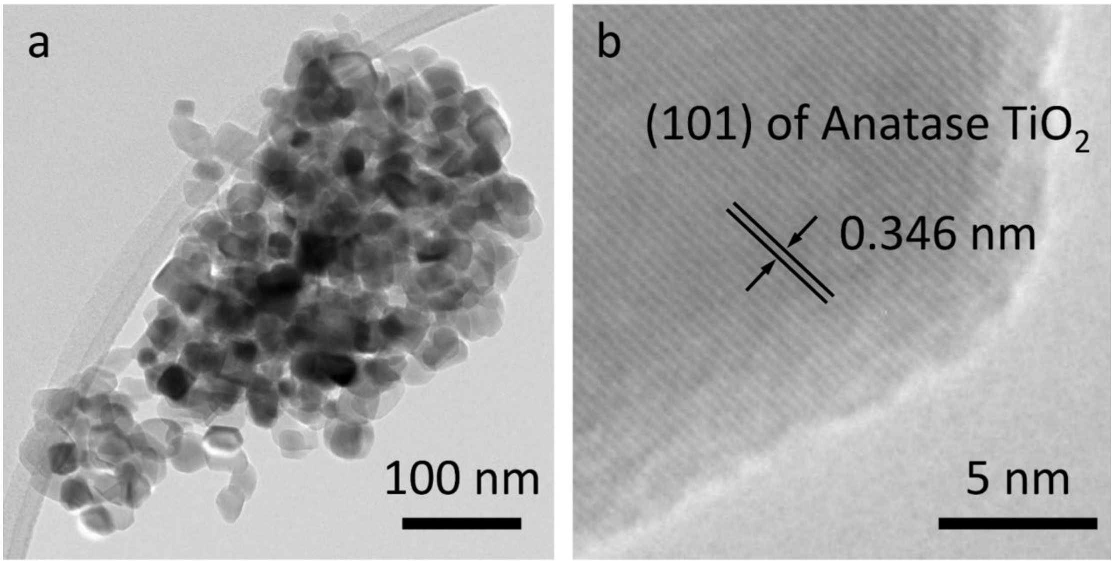

Figure S13. (a) TEM image of TiO_2; (b) HRTEM image showing (101) crystal planes of TiO_2.

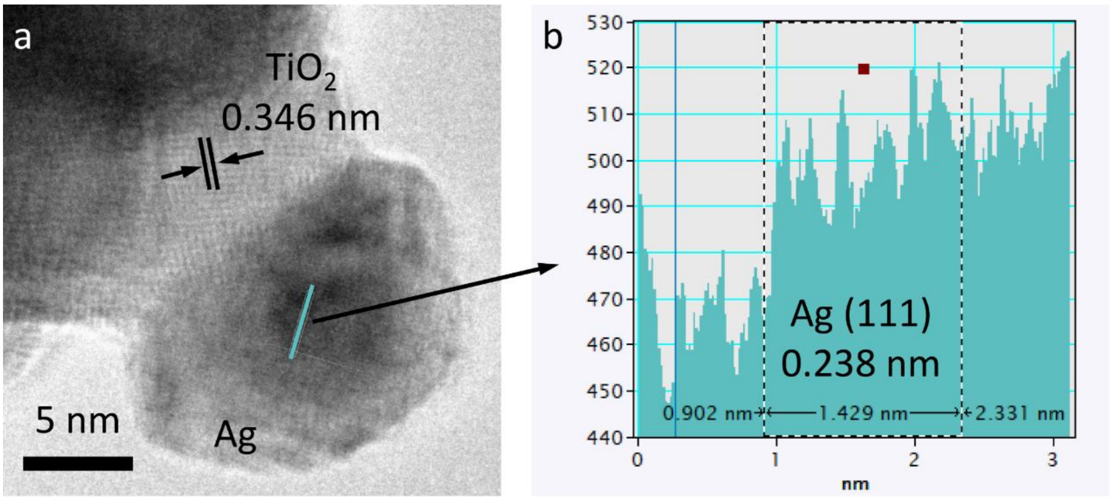

Figure S14. (a) HRTEM image of Ag/TiO_2, (b) line profiles showing (111) crystal planes of Ag on TiO_2, obtained from (a).

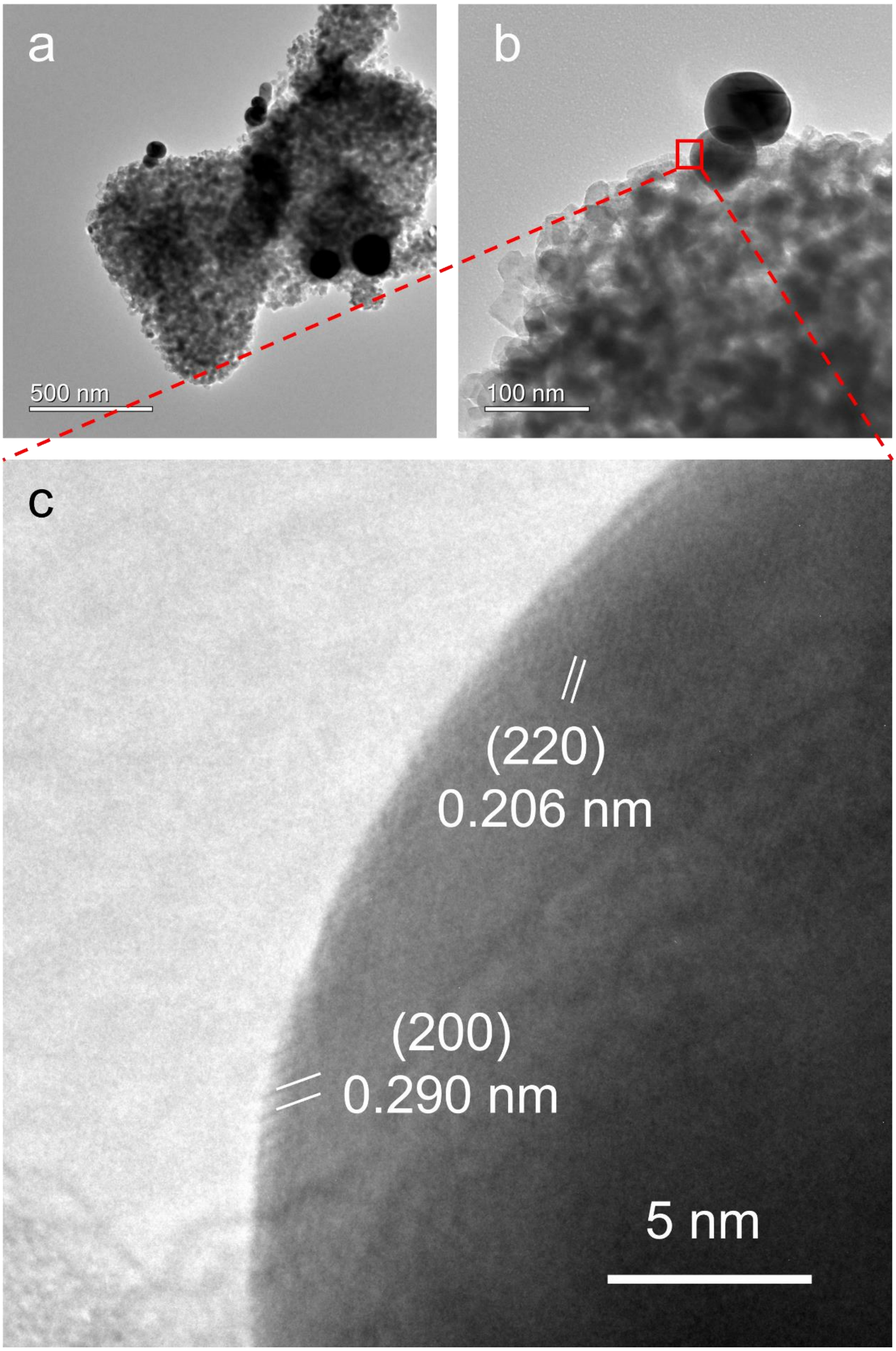

Figure S15. HRTEM images showing the existence of AgBr in Ag-AgBr/TiO_2.

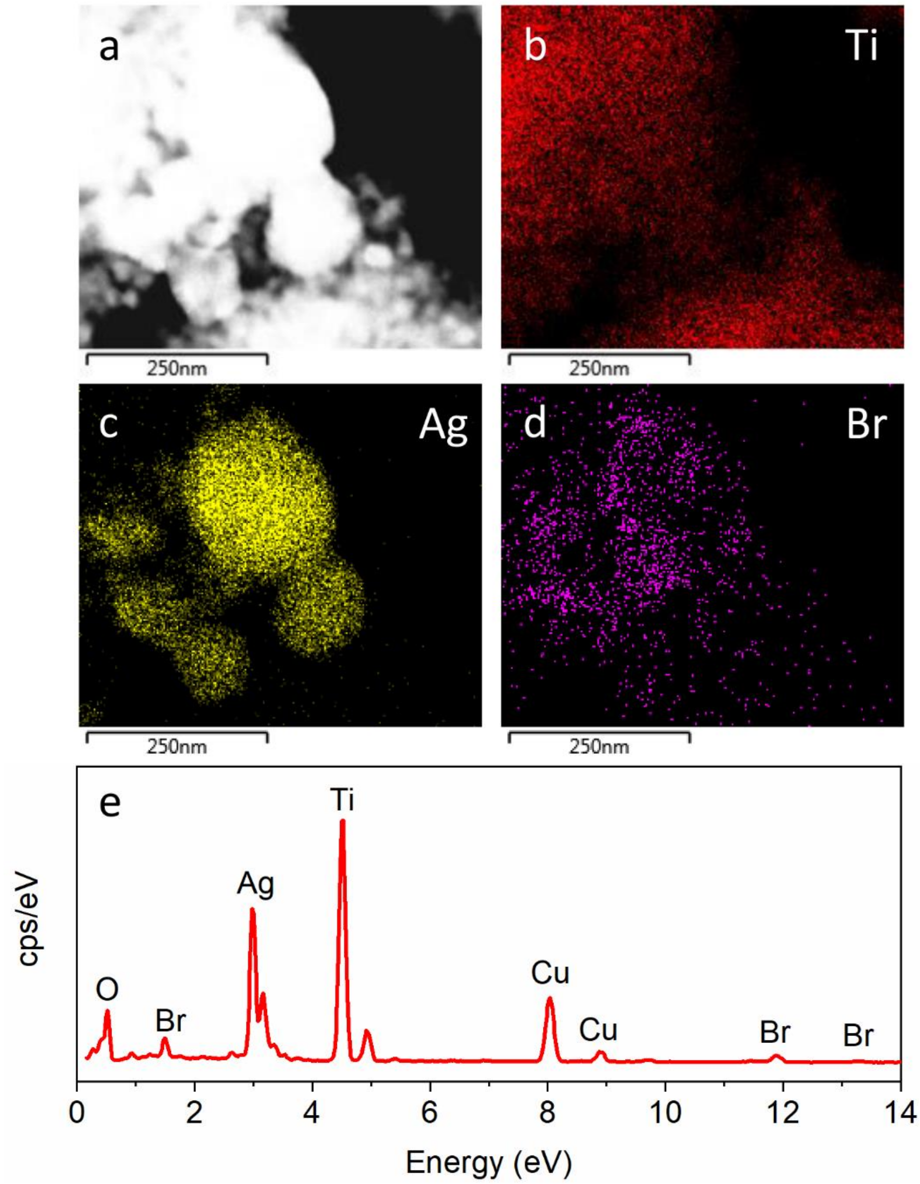

Figure S16. STEM-EDS mapping images of Ag-AgBr/TiO_2. (a) STEM image, (b-d) Ti, Ag, and Br mapping, (e) EDS sum spectrum.

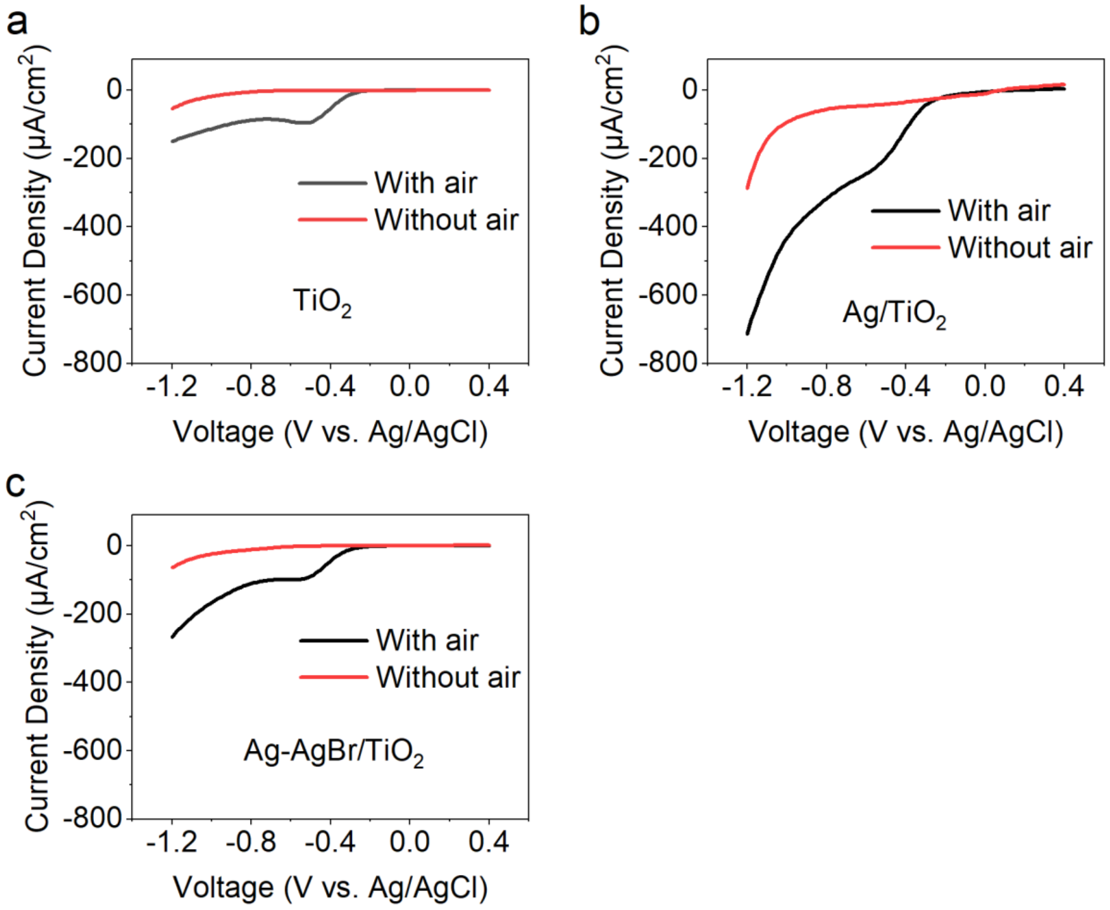

Figure S17. Electrochemical LSV spectra of (a) TiO_2, (b) Ag/TiO_2, and (c) Ag-AgBr/TiO_2 measured with and without air.

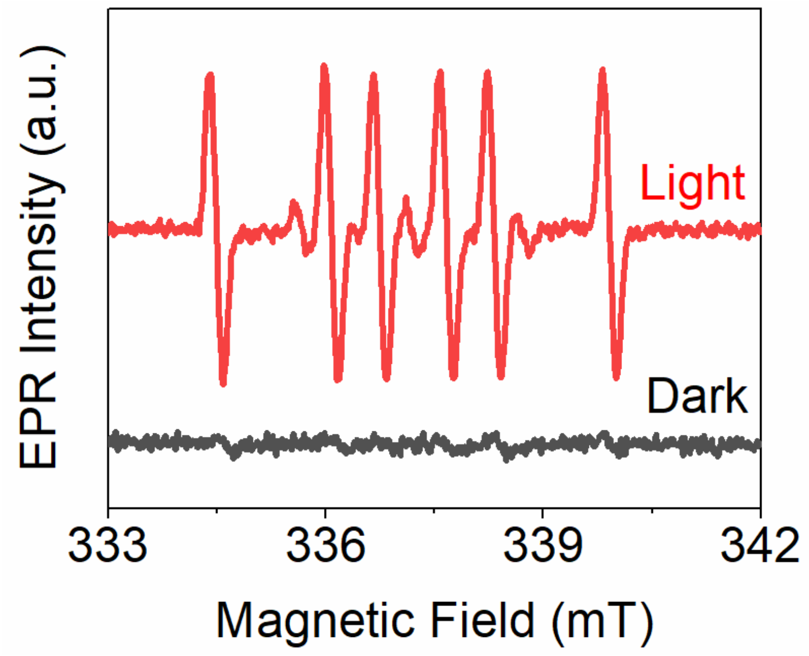

Figure S18. EPR O_2^- spectra of Ag-AgBr/TiO_2 under dark and light irradiation conditions.

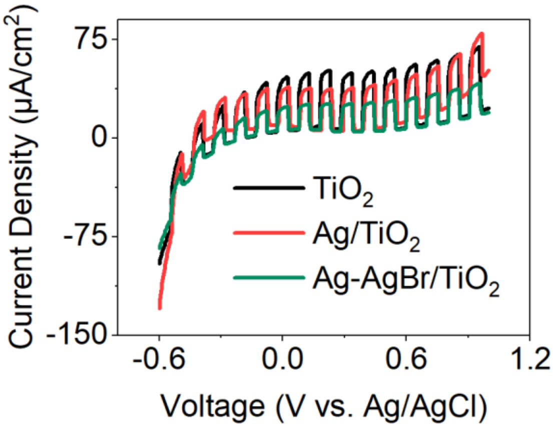

Figure S19. Transient photocurrent spectra of TiO_2, Ag/TiO_2, and Ag-AgBr/TiO_2 for methanol oxidation at a voltage window from -0.6 to 1.0 V vs. Ag/AgCl.

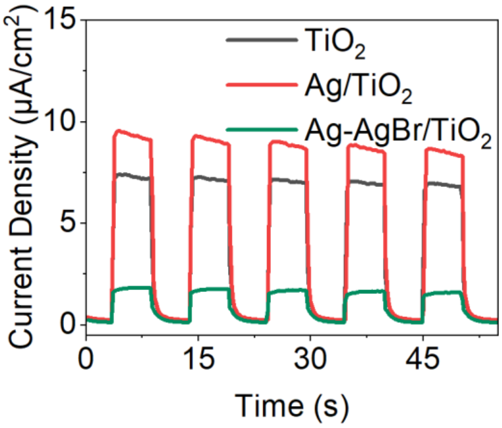

Figure S20. Photocurrent response of TiO_2, Ag/TiO_2, and Ag-AgBr/TiO_2 in a methanol-free electrolyte.

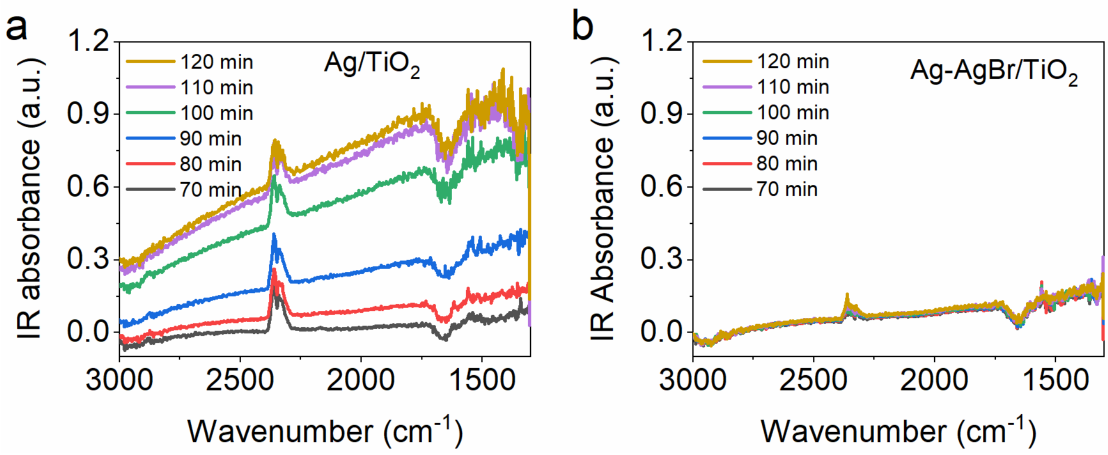

Figure S21. In situ DRIFTS spectra of Ag/TiO_2 and $Ag\text{-}AgBr/TiO_2$ under long-term light irradiation in reaction atmosphere (CH_4 : air = 40:1).

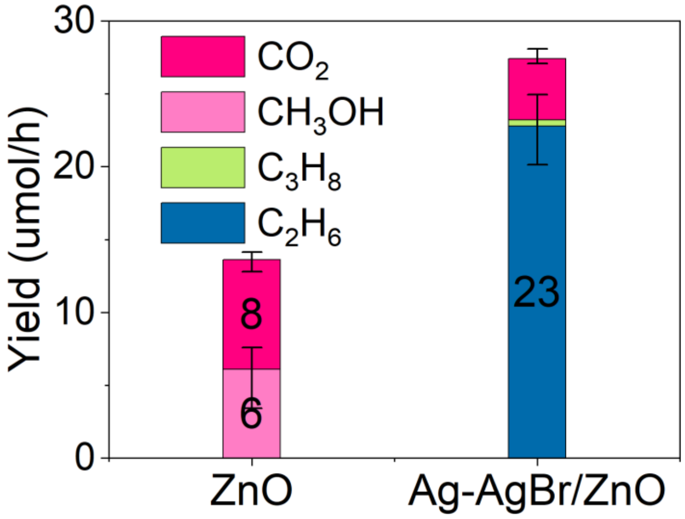

Figure S22. Photocatalytic OCM performance of ZnO and Ag-AgBr/ZnO. Reaction conditions: flow rate of CH_4, air, Ar = 40, 1, 359 mL/min, 6 bar, room temperature.

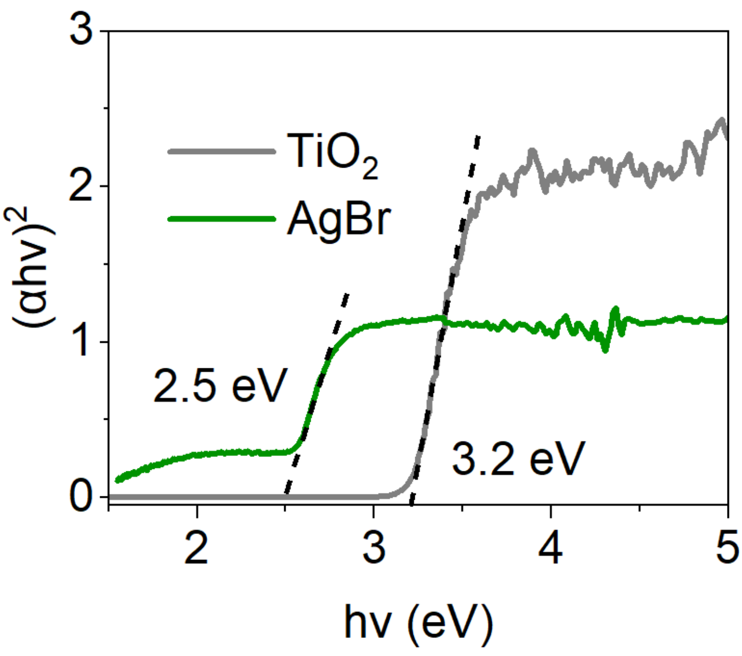

Figure S23. Kubelka–Munk plots showing the band gap of TiO_2 and AgBr.

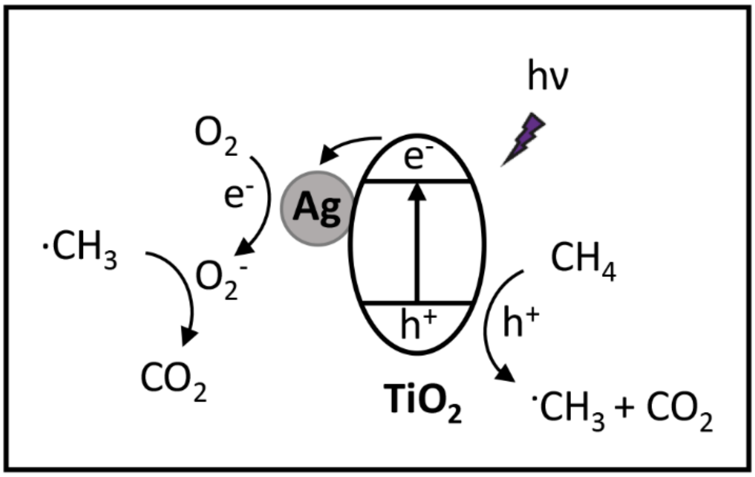

Scheme S2. A schematic illustration showing the photocatalytic reaction pathway of Ag-/TiO_2.

References

(1) Wu, X. Y.; Tang, Z.; Zhao, X.; Luo, X.; John Pennycook, S.; Wang, S. L. Visible-Light Driven Room-Temperature Coupling of Methane to Ethane by Atomically Dispersed Au on WO3. *J. Energy Chem.* **2021**, *61*, 195–202. https://doi.org/10.1016/j.jechem.2021.03.029.

(2) Wang, G.; Mu, X.; Li, J.; Zhan, Q.; Qian, Y.; Mu, X.; Li, L. Light-Induced Nonoxidative Coupling of Methane Using Stable Solid Solutions. *Angew. Chemie - Int. Ed.* **2021**, *60* (38), 20760–20764. https://doi.org/10.1002/anie.202108870.

(3) Yu, L.; Shao, Y.; Li, D. Direct Combination of Hydrogen Evolution from Water and Methane Conversion in a Photocatalytic System over Pt/TiO2. *Appl. Catal. B Environ.* **2017**, *204*, 216–223. https://doi.org/10.1016/j.apcatb.2016.11.039.

(4) Yu, L.; Li, D. Photocatalytic Methane Conversion Coupled with Hydrogen Evolution from Water over Pd/TiO2. *Catal. Sci. Technol.* **2017**, *7* (3), 635–640. https://doi.org/10.1039/c6cy02435a.

(5) Wu, S.; Tan, X.; Lei, J.; Chen, H.; Wang, L.; Zhang, J. Ga-Doped and Pt-Loaded Porous TiO2-SiO2 for Photocatalytic Nonoxidative Coupling of Methane. *J. Am. Chem. Soc.* **2019**, *141* (16), 6592–6600. https://doi.org/10.1021/jacs.8b13858.

(6) Yu, X.; Zholobenko, V. L.; Moldovan, S.; Hu, D.; Wu, D.; Ordomsky, V. V.; Khodakov, A. Y. Stoichiometric Methane Conversion to Ethane Using Photochemical Looping at Ambient Temperature. *Nat. Energy* **2020**, *5* (7), 511–519. https://doi.org/10.1038/s41560-020-0616-7.

(7) Yuliati, L.; Tsubota, M.; Satsuma, A.; Itoh, H.; Yoshida, H. Photoactive Sites on Pure Silica Materials for Nonoxidative Direct Methane Coupling. *J. Catal.* **2006**, *238* (1), 214–220. https://doi.org/10.1016/j.jcat.2005.12.002.

(8) Kato, Y.; Yoshida, H.; Satsuma, A.; Hattori, T. Photoinduced Non-Oxidative Coupling of Methane over H-Zeolites around Room Temperature. *Chem. Commun* **2002**, *51* (3), 223–231. https://doi.org/10.1016/S1387-1811(02)00268-8.

(9) Kato, Y.; Yoshida, H.; Satsuma, A.; Hattori, T. Photoinduced Non-Oxidative Coupling of Methane over Silica-Alumina and Alumina around Room Temperature. *Chem. Commun* **2002**, *51* (3), 223–231. https://doi.org/10.1016/S1387-1811(02)00268-8.

(10) Yoshida, H.; Matsushita, N.; Kato, Y.; Hattori, T. Active Sites in Sol-Gel Prepared Silica-Alumina for Photoinduced Non-Oxidative Methane Coupling. *Phys. Chem. Chem. Phys.* **2002**, *4* (11), 2459–2465. https://doi.org/10.1039/b111101a.

(11) Kato, Y.; Matsushita, N.; Yoshida, H.; Hattori, T. Highly Active Silica-Alumina-Titania Catalyst for Photoinduced Non-Oxidative Methane Coupling. *Catal. Commun.* **2002**, *3* (3), 99–103. https://doi.org/10.1016/S1566-7367(02)00056-0.

(12) Yoshida, H.; Chaskar, M. G.; Kato, Y.; Hattori, T. Active Sites on Silica-Supported Zirconium Oxide for Photoinduced Direct Methane Conversion and Photoluminescence. *J. Photochem. Photobiol. A Chem.* **2003**, *160* (1–2), 47–53. https://doi.org/10.1016/S1010-6030(03)00220-X.

(13) Yuliati, L.; Itoh, H.; Yoshida, H. Preparation of Isolated Highly Dispersed Titanium Oxides on Silica by Sol-Gel Method for Photocatalytic Non-Oxidative Direct Methane Coupling. *Stud. Surf. Sci. Catal.* **2006**, *162*, 961–968. https://doi.org/10.1016/S0167-

2991(06)81003-7.

(14) Yuliati, L.; Hattori, T.; Itoh, H.; Yoshida, H. Photocatalytic Nonoxidative Coupling of Methane on Gallium Oxide and Silica-Supported Gallium Oxide. *J. Catal.* **2008**, *257* (2), 396–402. https://doi.org/10.1016/j.jcat.2008.05.022.

(15) Li, L.; Cai, Y. Y.; Li, G. D.; Mu, X. Y.; Wang, K. X.; Chen, J. S. Synergistic Effect on the Photoactivation of the Methane C-H Bond over Ga 3+-Modified ETS-10. *Angew. Chemie - Int. Ed.* **2012**, *51* (19), 4702–4706. https://doi.org/10.1002/anie.201200045.

(16) Li, L.; Li, G. D.; Yan, C.; Mu, X. Y.; Pan, X. L.; Zou, X. X.; Wang, K. X.; Chen, J. S. Efficient Sunlight-Driven Dehydrogenative Coupling of Methane to Ethane over a Zn+-Modified Zeolite. *Angew. Chemie - Int. Ed.* **2011**, *50* (36), 8299–8303. https://doi.org/10.1002/anie.201102320.

(17) Yuliati, L.; Hamajima, T.; Hattori, T.; Yoshida, H. Highly Dispersed Ce(III) Species on Silica and Alumina as New Photocatalysts for Non-Oxidative Direct Methane Coupling. *Chem. Commun.* **2005**, No. 38, 4824–4826. https://doi.org/10.1039/b507698f.

(18) Yuliati, L.; Hamajima, T.; Hattori, T.; Yoshida, H. Nonoxidative Coupling of Methane over Supported Ceria Photocatalysts. *J. Phys. Chem. C* **2008**, *112* (18), 7223–7232. https://doi.org/10.1021/jp712029w.

(19) Yuliati, L.; Hamajima, T.; Hattori, T.; Yoshida, H. Highly Dispersed Magnesium Oxide Species on Silica as Photoactive Sites for Photoinduced Direct Methane Coupling and Photoluminescence. *Chem. Commun.* **2005**, No. 38, 4824–4826. https://doi.org/10.1039/b507698f.

(20) Meng, L.; Chen, Z.; Ma, Z.; He, S.; Hou, Y.; Li, H. H.; Yuan, R.; Huang, X. H.; Wang, X.; Wang, X.; Long, J. Gold Plasmon-Induced Photocatalytic Dehydrogenative Coupling of Methane to Ethane on Polar Oxide Surfaces. *Energy Environ. Sci.* **2018**, *11* (2), 294–298. https://doi.org/10.1039/c7ee02951a.

(21) Jiang, W.; Low, J.; Mao, K.; Duan, D.; Chen, S.; Liu, W.; Pao, C.-W.; Ma, J.; Sang, S.; Shu, C.; Zhan, X.; Qi, Z.; Zhang, H.; Liu, Z.; Wu, X.; Long, R.; Song, L.; Xiong, Y. Pd-Modified ZnO–Au Enabling Alkoxy Intermediates Formation and Dehydrogenation for Photocatalytic Conversion of Methane to Ethylene. *J. Am. Chem. Soc.* **2021**, *143* (1), 269–278. https://doi.org/10.1021/jacs.0c10369.

(22) Amano, F.; Akamoto, C.; Ishimaru, M.; Inagaki, S.; Yoshida, H. Pressure-Induced Dehydrogenative Coupling of Methane to Ethane by Platinum-Loaded Gallium Oxide Photocatalyst. *Chem. Commun.* **2020**, *56* (47), 6348–6351. https://doi.org/10.1039/d0cc01730b.

(23) Singh, S. P.; Yamamoto, A.; Fudo, E.; Tanaka, A.; Kominami, H.; Yoshida, H. A Pd-Bi Dual-Cocatalyst-Loaded Gallium Oxide Photocatalyst for Selective and Stable Nonoxidative Coupling of Methane. *ACS Catal.* **2021**, *11* (22), 13768–13781. https://doi.org/10.1021/acscatal.1c03786.

(24) Ishimaru, M.; Amano, F.; Akamoto, C.; Yamazoe, S. Methane Coupling and Hydrogen Evolution Induced by Palladium-Loaded Gallium Oxide Photocatalysts in the Presence of Water Vapor. *J. Catal.* **2021**, *397*, 192–200. https://doi.org/10.1016/j.jcat.2021.03.024.

(25) Lang, J.; Ma, Y.; Wu, X.; Jiang, Y.; Hu, Y. H. Highly Efficient Light-Driven Methane Coupling under Ambient Conditions Based on an Integrated Design of a

Photocatalytic System. *Green Chem.* **2020**, *22* (14), 4669–4675. https://doi.org/10.1039/d0gc01608j.

(26) Li, X.; Xie, J.; Rao, H.; Wang, C.; Tang, J. Platinum- and CuO x -Decorated TiO 2 Photocatalyst for Oxidative Coupling of Methane to C 2 Hydrocarbons in a Flow Reactor . *Angew. Chemie* **2020**, *132* (44), 19870–19875. https://doi.org/10.1002/ange.202007557.

(27) Song, S.; Song, H.; Li, L.; Wang, S.; Chu, W.; Peng, K.; Meng, X.; Wang, Q.; Deng, B.; Liu, Q.; Wang, Z.; Weng, Y.; Hu, H.; Lin, H.; Kako, T.; Ye, J. With Dioxygen. *Nat. Catal.* **2021**, *4* (December), 1032–1042.
